# Functional Optical Fiber Sensors Detecting Imperceptible Physical/Chemical Changes for Smart Batteries

**DOI:** 10.1007/s40820-024-01374-9

**Published:** 2024-03-18

**Authors:** Yiding Li, Li Wang, Youzhi Song, Wenwei Wang, Cheng Lin, Xiangming He

**Affiliations:** 1 Institute of Nuclear and New Energy Technology, Tsinghua University, Beijing, 100084 People’s Republic of China; 2 National Engineering Research Center of Electric Vehicles, Beijing Institute of Technology (BIT), Beijing, 100081 People’s Republic of China; 3 Shenzhen Automotive Research Institute of BIT (Shenzhen Research Institute of National Engineering Research Center of Electric Vehicles), Shenzhen, 518118 People’s Republic of China

**Keywords:** Smart battery, Advanced embedded optical fiber sensor, Battery internal physical/chemical state, Quality-reliability-life characteristic

## Abstract

The battery technology progress has been a contradictory process in which performance improvement and hidden risks coexist. Now the battery is still a “black box”, thus requiring a deep understanding of its internal state. The battery should “sense its internal physical/chemical conditions”, which puts strict requirements on embedded sensing parts. This paper summarizes the application of advanced optical fiber sensors in lithium-ion batteries and energy storage technologies that may be mass deployed, focuses on the insights of advanced optical fiber sensors into the processes of one-dimensional nano–micro-level battery material structural phase transition, electrolyte degradation, electrode–electrolyte interface dynamics to three-dimensional macro-safety evolution. The paper contributes to understanding how to use optical fiber sensors to achieve “real” and “embedded” monitoring. Through the inherent advantages of the advanced optical fiber sensor, it helps clarify the battery internal state and reaction mechanism, aiding in the establishment of more detailed models. These advancements can promote the development of smart batteries, with significant importance lying in essentially promoting the improvement of system consistency. Furthermore, with the help of smart batteries in the future, the importance of consistency can be weakened or even eliminated. The application of advanced optical fiber sensors helps comprehensively improve the battery quality, reliability, and life.

## Highlights

- Research progress of advanced optical fiber sensors in traction batteries and energy storage batteries is summarized.
- The embedded application mechanisms of different optical fiber sensors in batteries are discussed.
- Advanced optical fiber sensors adapting to batteries with diverse materials are reviewed.
- Advanced optical fiber sensors driving the development of future smart batteries are prospected.

## Graphical abstract

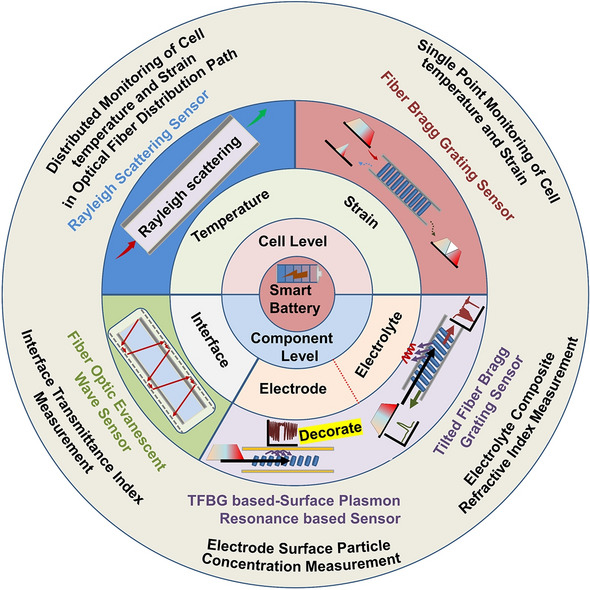

## Introduction

With the development of battery technology, lithium-ion batteries with high specific energy, long life, and high power are being increasingly widely used in electric vehicles or energy storage stations. For batteries, the state of charge (SOC), state of health (SOH) and state of safety (SOS) are strong bases for evaluating the working quality, working reliability and cycle life (QRL) [1]. With the continuous operation of lithium-ion batteries, frequent safety accidents have cast a shadow on their development.

At this stage, the development of intrinsically safe anodes, cathodes and electrolytes is in the “bottleneck period” [2], and solving the battery safety problem is urgent. Therefore, improving battery safety through battery self-monitoring and self-management has become an effective method, spawning an extremely important scientific branch—*smart batteries*. Different from traditional battery intelligence at the module level or system level, the focus for smart batteries is on improving the intelligence of a single battery to enable it to have self-monitoring and self-management capabilities, which is now becoming one of the mainstream research hotspots. The European Union (EU) has made a long-term plan for the development of smart batteries in the technical blueprint of *Battery 2030*+ *Roadmap* [3] released in 2020. Taking electric vehicles as an example, the important “big three parts” are the motors, controllers and batteries, the first two of which have been or can be intelligentized at any time [4, 5], while the intelligentization of batteries, especially at the cell level, as the last “plank” of the “big three parts” intelligentization, needs to be focused on. This is mainly because the systems are composed of a large number of single batteries; one aging or defective battery will degrade the system performance or directly threaten the system safety, while a smart battery at the cell level makes consistency less important, and the battery configuration can be flexible [6].

The development of high-safety, long-life and, high-performance smart lithium-ion batteries (SLIBs) is inseparable from research on the working and aging mechanisms of batteries. Understanding the application principle of observation with advanced sensors embedded in batteries and the impact on the battery performance is also urgent.

During the long service life of a battery, due to the repeated insertion and removal of lithium ions into/from electrodes and the resulting heat generation phenomenon, the battery will experience periodic expansion and contraction, which means that the battery has a kind of “breathing effect”. First, this “breathing” phenomenon generates mechanical stress in the battery, and second, long-term repeated expansion-contraction will produce a “stress fatigue” effect, damaging the active material. In addition to the periodic expansion-contraction, the battery aging, such as thickening of the solid electrolyte interphase (SEI) and decomposition of the electrolyte, will cause abnormal expansion of the battery in the out-of-plane direction [7], which aggravating the mechanical stress fatigue effect.

In some extremely harsh environments, such as in low temperatures, the Li^+^ intercalation potential (~0.1 V vs. Li/Li^+^) of the graphite anode is close to 0 V, and the graphite electrode surface will undergo severe lithium plating [8]. The lithium plating will sometimes not be in contact with the electrode, and the lithium metal covered by the passivation film will drift into the electrolyte and form “dead lithium” [9]. In addition, low temperature will accelerate the deposition/decomposition of electrolyte mixture, forming a thick and loose interfacial film [10]. However, at high temperatures, lithium-ion batteries will have more unexpected side reactions, which will produce side reaction particles [11]. In addition, the frequent “breathing” effect makes the active material have a non-negligible lattice variation or uneven evolution of the battery structure [12, 13], so the weakened lattice and the deteriorated structure lead to defects [14], such as particle breakage and pulverization of the active material at the micro-level. With the improvement of the battery management system (BMS), the frequent overcharge and over-discharge in the past have been significantly curbed. However, due to the very large number of batteries in a system, micro-overcharge (under normal temperature conditions, one or several overcharges or overcharge to a certain voltage, in which the lithium-ion battery will not fail or thermal runaway) of local batteries is inevitable. The micro-overcharge will lead to excessive release of lithium ions from the positive electrode, leading to collapse of the structure and causing the transition metal to dissolve [15].

High precision mechanical, thermal, and electrochemical performance and gas [16] measurements of batteries, such as expansion force, expansion displacement, temperature, high-precision Coulombic efficiency, material degradation, combustible gas, can indicate imperceptible parasitic reactions, side reactions, and even thermal runaway. These measurements help predict the actual working state, safety state, and battery life. However, the imperceptible physical/chemical changes in the internal core of the battery, as well as micro-damage to the battery, are still difficult to accurately understand [17].

From the above analysis, at the macro-level, the expansion-contraction of battery volume and the temperature changes can be observed through pressure sensors [18], strain sensors [19], thermocouples [20–22], etc. Nevertheless, due to the morphological characteristics and measurement principles of these sensors, they can generally only measure the external state change of the battery and cannot obtain internal information of the battery. With the improvement of the battery energy density [23], application of new electrode materials [24, 25] and demand for upgrading battery technology, there is an urgent need to identify the performance of electrode materials, electrolytes and electrode interfaces inside batteries. Some technologies that can obtain the internal state information of a battery, such as ultrasonic detection [26, 27], optical color contrast [28–31], and electrochemical window infrared detection technology [32, 33]. These technologies have been applied to a certain extent at the laboratory level. However, these methods have large detector components, which are not suitable for embedded installation and mainly focus on point measurement, or require opening of a transparent window into the battery and use of professional color recording and analysis software. They have a certain application value for a single battery, but there is the defect of great difficulty in application at the module level. With the progress of technology, optical fiber sensors are being increasingly widely used in batteries, and their radial dimensions are usually 100 μm or less (excluding coating layer) [34], which will not cause substantial damage to the battery structure. An optical fiber made of silicon dioxide is stable in nature and can work in a harsh electrolyte environment for a long time without affecting the battery performance [35, 36]. Due to the measurement characteristics of optical fibers, in theory, a single optical fiber can measure the status of multiple batteries at the same time, of course, multiple measurement points on one optical fiber can achieve multipoint measurement within one battery. The optical fiber sensing system formed by the layout of multiple fiber optic arrays can achieve large-scale deployment of online battery monitoring. This technical effect has been achieved in reality [37]. Relying on various measurement principles of optical fiber sensors, such as vertical gratings [38, 39], tilted gratings [40], evanescent waves [41–43], surface plasmon resonance [44, 45], Rayleigh scattering [39, 46] and other effects, real-time in-situ measurement of battery internal core key features, such as the stress-strain [47], temperature [48–51], electrolyte refractive index [49, 52, 53], SEI growth [36, 54], electrode material redox process [41] and ion concentration [42], can now be achieved. This is crucial for understanding the electrode interface dynamics, electrolyte degradation, battery capacity attenuation, and evolution of the electrode material phase and structure. SLIBs prepared by using advanced embedded optical fiber sensors are conducive to clarifying the battery internal processes, thus helping in achieving progress in battery technology.

**Fig. 1:**
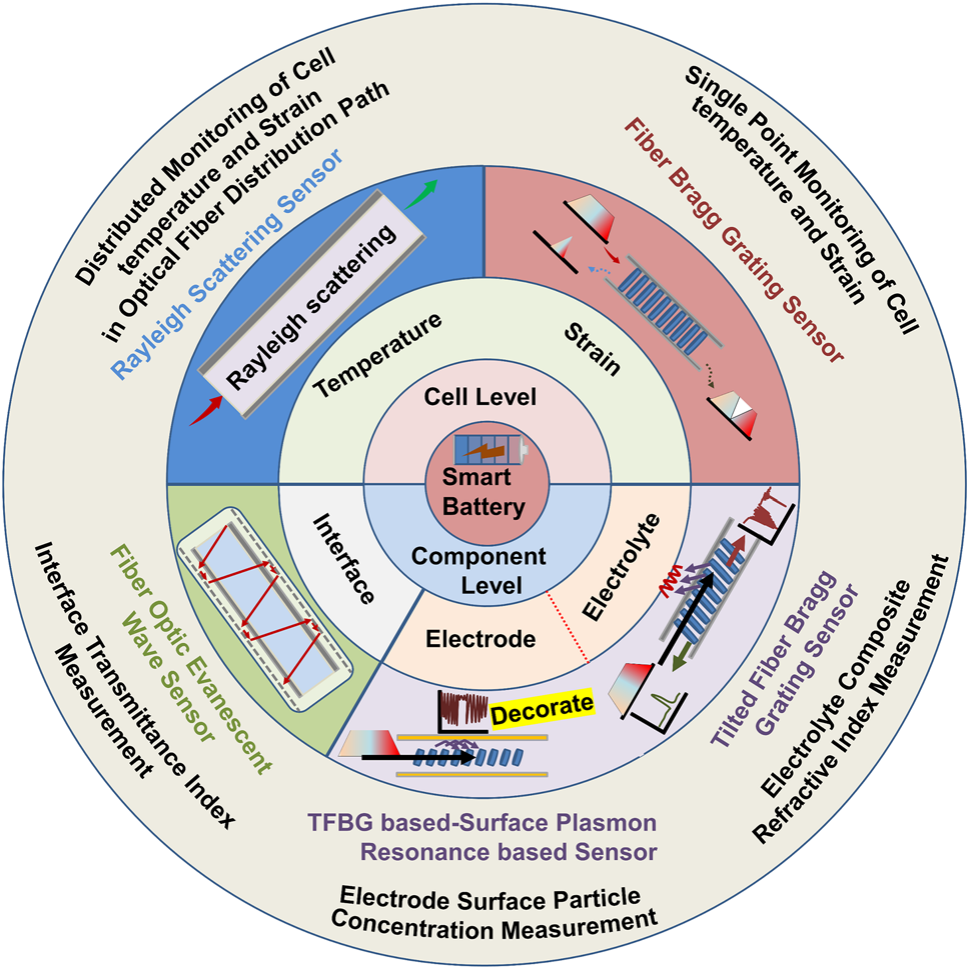
Promising optical fiber sensors in battery applications

Against the backdrop of current SLIB research and development, this paper summarizes and evaluates the embedded sensor technology required in the first stage (first stage: develop multisource sensing technology, and achieve transparent to the battery chemical environment. Three stages can be seen in Ref. [3]) and mainly reviews and comments on the theory and application of advanced optical fiber sensors in understanding the internal mechanisms of a battery, as shown in Fig. 1. This paper reviews the in-situ online observation of the core reaction mechanisms in a battery using optical fiber sensors and proves that the battery interior can be elucidated by employing advanced optical fiber sensors. To the best of our knowledge, most of the current review studies on the application of optical fiber sensors in batteries concentrate on the sensor working mechanism, simple monitoring processes, and monitoring results. It is unclear how optical fiber sensors can monitor which key processes and achieve what results. Regarding this, this paper reviews and studies the insight ability and future development of quasi-distributed optical fiber sensors, such as the fiber Bragg grating (FBG) sensor, tilted fiber Bragg grating (TFBG) sensor, fiber optic evanescent wave (FOEW) sensor, surface plasmon resonance-based (SPR) optical fiber sensor (the above point-measurement optical fiber sensors are set as quasi-distributed sensor; this is because, as mentioned earlier, multiple measurement points can be fabricated on one single fiber to achieve quasi-distributed measurement) and distributed optical fiber sensor based on the Rayleigh scattering effect in monitoring the core physical/chemical state of lithium-ion batteries and other promising energy storage devices.

## Promising Optical Fiber Sensors in Future SLIBs

At present, the development of lithium-ion batteries is still in the “original” stage. These batteries only have basic charging and discharging functions. The use of advanced optical fiber sensors can help a battery “sense” itself. This section summarizes quasi-distributed optical fiber sensors with broad application prospects in batteries in the future, such as FBG sensors, TFBG sensors, FOEW sensors, SPR-based optical fiber sensors and distributed optical fiber sensors-for example, optical fiber sensors based on the Rayleigh scattering principle. It should be noted that current point measurement-based optical fiber sensors have the capability of multiplexing, meaning that multiple measurement points can be connected in series through one single fiber. In practical applications, using a single point measurement fiber is, in fact, giving up the advantages of optical fiber sensors. Therefore, in this section, the point-measurement optical fiber sensors are classified as the quasi-distributed sensor. This part focuses on describing the effects that different optical fiber sensors can achieve in batteries. The specific working mechanisms and mathematical models of optical fiber sensors can be obtained in the vast majority of relevant literatures. This part will not provide too much description for these.

### FBG Sensor: Battery Routine Mechanical-Thermal Monitoring

**Fig. 2:**
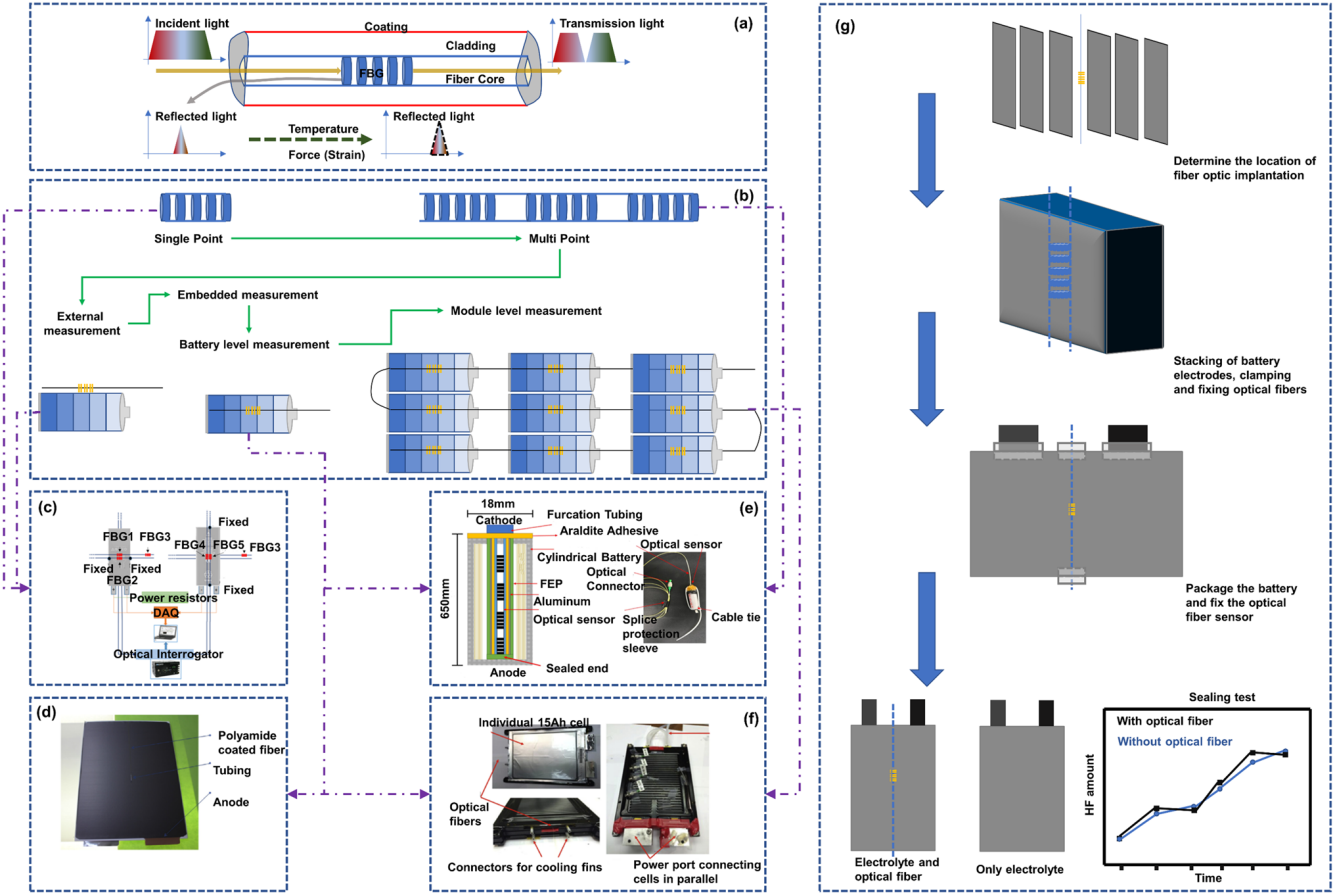
Application of FBG sensors to measure the cell strain-temperature at the laboratory level and before industrialization. **a** FBG sensor structure and action mechanism. When the battery is loaded with temperature or strain, the FBG grid spacing changes, and the central wavelength of reflected light shifts. The central wavelength shift is linearly related to the temperature and strain. **b** The evolution of FBG in battery applications. From single point to multipoint, from external to internal, and from single battery to module even system. **c** Bidirectional strain measurement of an FBG sensor in a single cell. Reproduced with permission from Ref. [56]. Copyright 2018 MDPI. **d** Embedded FBG sensor core temperature measurement. Reproduced with permission from Ref. [35]. Copyright 2018 Elsevier. **e** Embedding of an FBG in a large pouch battery. Reproduced with permission from Ref. [55]. Copyright 2017 Elsevier. **f** Traction battery pack with an FBG embedded in the core. Reproduced with permission from Ref. [35]. Copyright 2017 Elsevier. **g** The process of embedding optical fibers in batteries involves the steps of implantation position determination, stacking fixation, encapsulation sealing, and testing in sequence. Reproduced with permission from Ref. [35]. Copyright 2017 Elsevier

FBG sensors, due to their high monitoring precision and fast detection speed, are well-suited for observing changes in the mechanical and thermal behavior of batteries during charging and discharging processes. The variations in these physical characteristics are closely related to the ion transport, lithium ion insertion/extraction, and electrochemical reactions inside the battery, making FBG sensors an excellent tool for monitoring the evolution of battery states. As the optical fiber sensor with the most concise application structure, most mature technology, and lowest cost, the FBG sensor, as shown in Fig. 2a, has a large number of applications and research achievements at the laboratory level [55, 56]. In the industry, before large-scale industrialization, relevant products were also tested [35, 57]. This process has gone through the transition from single point FBG to multipoint FBG, from external measurement to embedded measurement, and from a single battery to module and even system-level progress, as shown in Fig. 2b–f. Generally, an FBG sensor is mostly used to measure the temperature and electrode (cell) expansion inside or outside the battery by using its property of being sensitive to temperature and strain at the same time.

When the battery experiences electrode expansion and temperature rise due to lithium ion insertion/removal and the redox reaction [58], the grid spacing of the FBG attached to the electrode surface will change with the battery expansion or temperature rise, and the peak of the reflected light will accordingly shift. As shown in Fig. 2a, the optical physical quantities of the FBG and temperature-strain can be correlated by reading the position of the reflected light peak [59, 60]. Of particular interest is that in an FBG, the temperature and strain show a highly linear relationship with the change in the peak position, which provides great convenience in using the FBG to easily measure the battery temperature and strain and realize decoupling of the two features. Currently, with the maturity of the fiber manufacturing process and grating etching process, the cost competitiveness of FBGs [61] meets the needs of large-scale industrial applications. Compared to mature and cost-effective optical fibers, the cost of another crucial component of optical fiber sensors—the modulator–demodulator (modem), is currently a significant barrier preventing the widespread application of FBG. The cost of advanced modems typically ranges around CNY 100,000. Placed within the context of electric vehicles, this cost is comparable to the entire battery system cost. Such an expensive modem would significantly increase the overall cost of the relevant energy systems, hindering their widespread adoption. However, with technological advancements, the current size of the simplest laser source emission systems can be reduced to the dimensions of several stacked coins. In the future, if FBG sensor systems are integrated into relevant energy storage systems, the functions of the modem can be progressively decentralized. The laser source system can selectively use a light source with a specific fixed window spectrum, and the receiving and processing modules of the modem can be integrated into the battery management system (BMS). The traditional modem’s auxiliary and heat dissipation modules can be shared with the BMS. In this configuration, the costs associated with relevant hardware and software can be leveled out through mass production. FBG sensors integrated into energy storage systems in the future will be as simple and cost-competitive as traditional sensors. Therefore, the FBG, as a representative of small-size, high-stability and multipurpose optical fibers, may be the most promising optical fiber sensor in the future [52, 62].

Among the many advantages of FBG, an important issue that cannot be ignored is the force-temperature coupling behavior. That is to say, when FBG is subjected to battery temperature and external force, both of them will simultaneously cause displacement of the FBG center wavelength. How to decouple force-thermal parameters is a key issue. Currently, the conventional solution is the dual-FBG-decoupling method, where two FBGs with different force-thermal parameters are used to simultaneously measure the battery, or one FBG is fixed, while the other FBG is released to make it only sensitive to temperature, and then the relevant parameters can be decoupled by solving a simple one-dimensional quadratic equation system; Secondly, based on simple mathematical principles, knowing one unknown quantity can solve another unknown quantity. Usually, a thermocouple or strain gauge can be attached next to the FBG to solve another unknown quantity with known temperature or strain; In addition, decoupling can also be achieved for additional geometric structures of FBG, such as arranging the FBG up and down, causing the mechanical vector of FBG to change in the opposite direction, or using the thermal expansion and contraction effects of the structure to artificially eliminate changes caused by temperature effects. All of the above methods can achieve decoupling measurement of FBG force-temperature parameters, promoting the application of FBG in in-situ monitoring of batteries.

A key aspect for the stable operation of optical fibers in batteries is the correct embedding of the fibers inside the battery, as illustrated in Fig. 2g. Taking the embedded application of FBG optical fibers in pouch batteries as an example. One initial consideration is the embedding process. Typically, the optical fibers are embedded between the electrode sheets before battery assembly, with the position generally at the geometric center of the electrode. Subsequently, the optical fibers are stacked and clamped between the electrode sheets to achieve initial fixation. Then, the battery jellyroll is placed within the aluminum-plastic film for positioning. The second critical aspect is sealing. A research team used materials consistent with the heat-sealing glue for sealing electrode tabs to envelop the optical fibers. This ensures fixation at both ends of the aluminum-plastic film, achieving the internal fixation of the optical fibers within the battery. Subsequent comparative testing is conducted on batteries with and without embedded optical fibers, using the HF content as an indicator. By recording the penetration of moisture and the HF generated by the reaction with the electrolyte, the sealing performance of the two encapsulation methods is compared. The results show that after 4 weeks, the embedding of optical fibers did not compromise the sealing integrity of the battery [35] .

Another crucial consideration in the application of FBG sensors in batteries is whether the sensor can maintain long-term stability and proper functionality within the battery. This is fundamental for the sensor to effectively monitor the battery’s status without interfering with its normal operation. Regarding the former, Raghavan et al. [35] discovered that FBGs made of silicon dioxide material, when coated with a special coating, can fully withstand the electrochemical effects of daily battery use. On the one hand, properly coated FBGs can withstand high temperatures ranging from 200 to 800 °C (a temperature range far exceeding the normal operating temperature of batteries and even surpassing certain characteristic temperatures during battery thermal runaway). On the other hand, the optical fibers can endure the stretching effects caused by changes in battery volume, allowing them to operate within the elastic region to ensure a highly linear response. Also, Raghavan et al. [35] comparing batteries with embedded FBGs to control batteries, found that both exhibited a capacity decline rate of less than 20% after more than 1100 and 1300 cycles, respectively. The Coulombic efficiency for both scenarios exceeded 0.999, demonstrating that the batteries with embedded FBGs did not significantly impact battery performance. Similar validation was performed by Huang et al. [36], who found no significant differences in cycling capacity and rate performance between batteries with embedded FBGs and control batteries over at least 100 cycles.

### TFBG Sensors: Electrolyte Internal Environmental Monitoring

**Fig. 3:**
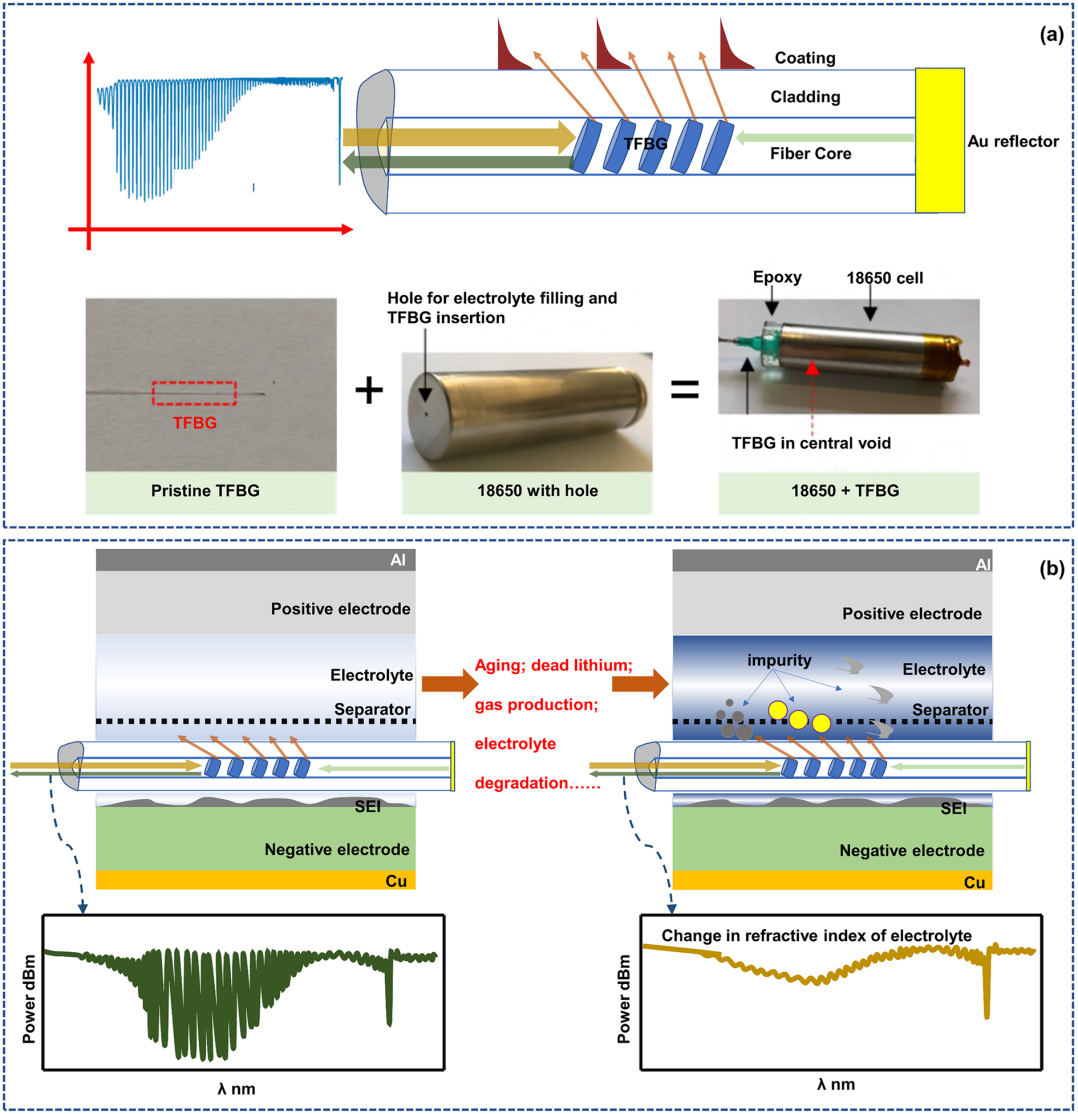
TFBG monitoring of the electrolyte environment. **a** Working mechanism and application scenario of the TFBG in batteries. When the incident light enters the fiber and reaches the grating, the light meeting the Bragg condition will undergo Bragg reflection, while the light meeting the conditions of a certain order cladding radiation mode will be coupled into the cladding and exchange energy with the external environment, realizing monitoring of the surrounding electrolyte environment. Reproduced with permission from Ref. [49]. Copyright 2021 Royal Society of Chemistry. **b** The evolution of electrolyte refractive index with battery health state in TFBG Wavelength. The suspended dead lithium, gas production, and degradation of the electrolyte’s performance caused by battery aging can all lead to significant changes in the comprehensive refractive index of the electrolyte detected by TFBG, which is reflected in the mode conversion of various modes in TFBG, including the center position of the mode peak and the power of the peak. Reproduced with permission from Ref. [49]. Copyright 2021 Royal Society of Chemistry

For a traditional FBG, the grating is perpendicular to the fiber axis. When a special manufacturing process is used to tilt the grating direction and axis to a certain angle, a tilted FBG sensor is obtained, as shown in Fig. 3a. In this case, the reflected light will not strictly reflect back to the incident end; instead, the reflected light is transmitted to the cladding and then coupled with the external environment. When a TFBG is embedded into the battery, it interacts with the electrolyte environment through the reflected light from the overflowing optical fiber, acting like a “nerve”, constantly monitoring the internal environment of the battery’s “body fluid” (electrolyte). The reflected light spectrum will exhibit a different mode from that of the FBG. There are many coupling modes in a TFBG, and light from the core can be coupled as follows: first, a subset of the large number of modes can be guided by the (much larger) cladding of the fiber; second, leaky modes of the cladding arise; and then, highly directional light beams even radiate out of the fiber [40].

The light meeting the conditions of a certain order cladding radiation mode will be coupled into the cladding and exchange energy with the external environment, so the external environmental state can be found from the spectrum. After long cycling, a battery will experience excessive consumption or side reaction of the electrolyte, leading to the appearance of micro-solid particles in the electrolyte [49]. The lithium will be coated by an insulating passivation film and separate from the electrode, becoming “dead lithium” suspended in the electrolyte [63]. The consumption of the electrolyte itself and these substances floating in the electrolyte will alter the chemical composition of the battery itself, consequently causing changes in the optical signal sensed by TFBG. Thus, the TFBG sensor can easily “perceive” the turbidity process of the electrolyte by measuring the change in the refractive index caused by these phenomena (Fig. 3b). Because the Bragg mode and cladding mode are sensitive to the temperature and refractive index [64, 65], respectively, and there is no cross-sensitivity between them, the TFBG sensor has significant advantages in eliminating interference between temperature sensing and “seeing” substantial changes in the electrolyte.

Through the analysis of a case, it can be found that the TFBG has a unique advantage in sensing changes in the battery electrolyte environment. The reason why TFBG can achieve this unique monitoring effect is mainly based on its core-cladding mode coupling. TFBG uses a special process to tilt the grating, thereby introducing dozens or even hundreds of narrow linewidth cladding mode excitations, while retaining the backward conductive core mode that meets the Bragg condition. This allows these cladding modes to have rich mode field distributions and different response characteristics to the environment. The consumption of electrolyte inside the battery or energy storage device, the detachment of active materials, and the dissociation of “dead Li” may trigger different coupling modes. In the future, with the progress of pattern recognition technology, different aging and failure paths of the battery may be understood through TFBG. In addition, TFBG can achieve other unexpected effects through special additional processes by using techniques such as fiber taper and staggered fusion. TFBG can monitor the vibration, bending, and other environments of energy storage devices, combined with materials such as liquid crystals and magnetic fluids. TFBG can even monitor electric and magnetic fields, which may be an effective way to monitor micro-internal short circuits (electronic current induced magnetic field fluctuations in batteries) in the future. The extremely narrow spectral bandwidth of TFBG provides ultra-high detection sensitivity and resolution for its sensing. Combined with SPR technology, TFBG can achieve precise monitoring of important electrochemical processes in batteries, which will be detailed in the following part. In terms of cross-sensitivity decoupling, by referring to the wavelength and power level of the TFBG backward reflection fiber core mode, the inherent temperature cross-sensitivity problem in the sensing measurement process is completely eliminated, which greatly improves the accuracy and stability of the sensor. The embedded operando monitoring application of TFBG in batteries or energy storage devices will have great development space.

TFBG sensors and FBG sensors do not have essential differences in material and composition. The most notable structural difference between the two lies in the tilt angle of the internal grating. While the grating of FBG sensors is perpendicular to the axial direction, TFBG sensors have a certain tilt angle with respect to the axial direction. This tilt angle depends on the manufacturing process and the mode of interest to the researcher. The embedding form and corrosive environment faced by TFBG in batteries are similar to those of FBG. Referring to the working conditions and environments of FBG, it can be inferred that TFBG generally ensures long-term stability in batteries. Additionally, similar to the grating of FBG, TFBG’s grating has protection in the form of a fiber core and coating layer on the outer side. The influence of the battery environment on the grating can be neglected, and therefore, the grating’s ability to reflect incident light will not be significantly affected. In this regard, Liu et al. [66] conducted cycles of over 400 hours on TFBG in Li-S batteries. The experimental results showed that TFBG maintained good wavelength stability with high repeatability and minimal impact on the battery’s performance. This preliminary evidence suggests the potential for stable operation of TFBG in batteries. However, the long-term performance degradation and changes of TFBG after prolonged operation throughout the lifecycle of the battery remain one of the key research issues. This is crucial for determining whether the sensor can effectively perceive changes in the battery’s internal environment over time.

### FOEW & TFBG-SPR Sensors: Electrode Interface Monitoring

**Fig. 4:**
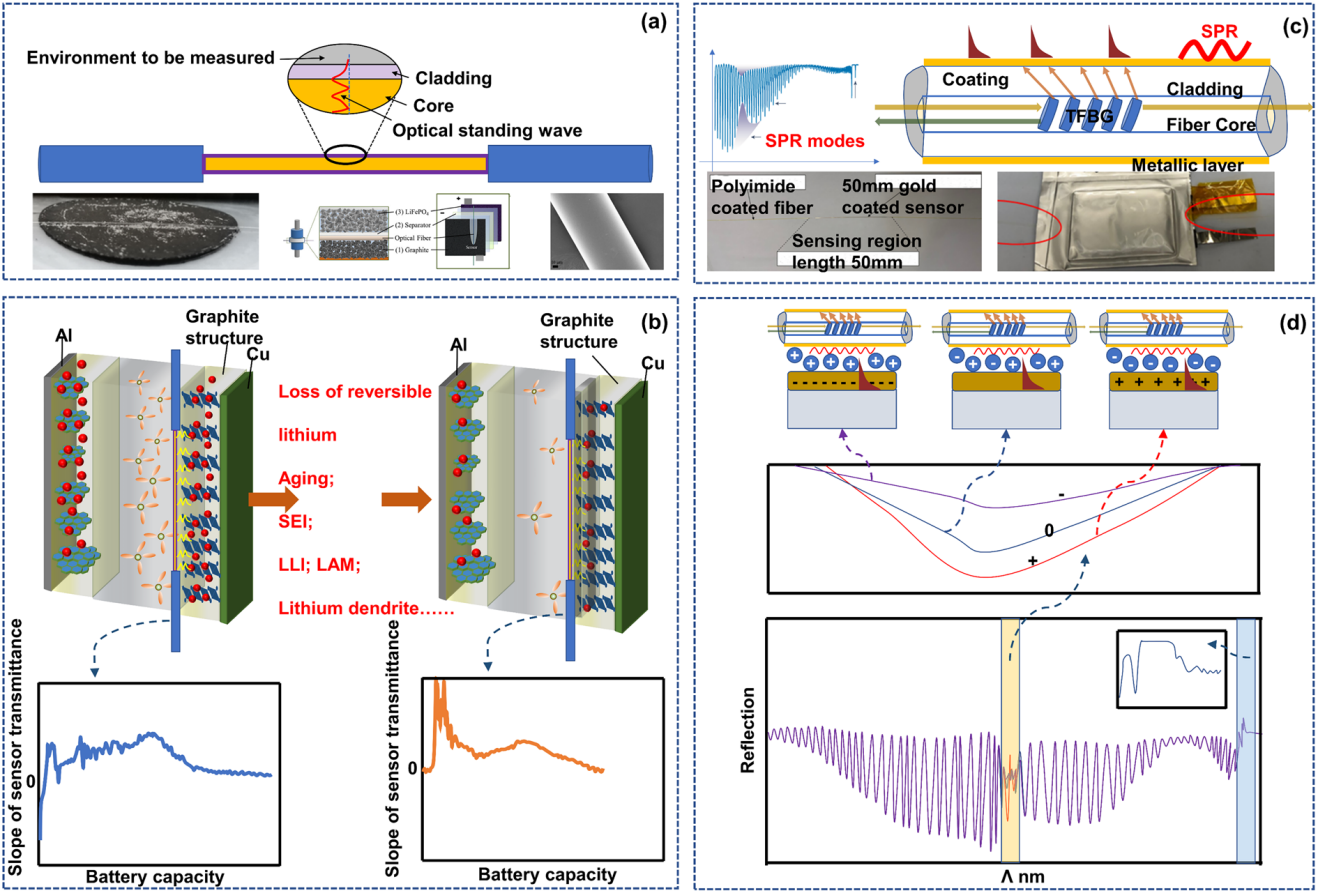
FOEW sensor and SPR sensor for battery surface monitoring. **a** FOEW sensor: The evanescent wave effect occurs when a light wave is incident from a dense medium to a light sparse medium, and total reflection occurs. The evanescent wave is a surface wave. Its amplitude exponentially decreases with increasing depth perpendicular to the interface, and the phase changes with the tangential direction. Reproduced with permission from Ref. [70]. Copyright 2017 ACS Publications. Ref. [71]. Copyright 2022 ACS Publications. **b** The loss of reversible lithium leads to changes in the detectable optical properties of FOEW. The optical transmissivity of the evanescent wave is related to the lithium-ion concentration on the surface of the graphite particles, when the capacity of the lithium-ion battery declines after long cycling due to the reduction of recyclable graphite lithium content, the FOEW transmission amplitude decreases. Reproduced with permission from Ref. [42]. Copyright 2020 Elsevier. **c** SPR sensor: Total internal reflection of incident light occurs at the interface between an optical fiber and a metal film, and the evanescent wave causes regular oscillation of metal surface electrons at the interface between the metal film and medium, which excites a surface plasmon wave. When the incidence angle or wavelength is a certain value, the wave vectors of the surface plasmon wave and evanescent wave are equal along the interface between the metal film and medium such that the wave vectors match, and the two resonate. The incident light is coupled with the surface plasmon wave through the evanescent wave, and the energy is strongly absorbed by the metal surface electrons, which makes the reflected light energy sharply drop and generates the surface plasmon resonance phenomenon. Reproduced with permission from Ref. [44]. Copyright 2022 MDPI. **d** The changes in ion concentration and state on the electrode surface affect the SPR waveform. The SPR mode is closely related to the electrode surface polarity, and as the potential gradually becomes positive, the SPR wave becomes deeper. At the same time, the core mode is not related to the polarity, and research shows that it is only related to temperature and only changes with temperature. Reproduced with permission from Ref. [45]. Copyright 2018 Springer Nature

The TFBG mentioned in the previous section can perceive the electrolyte environment around optical fibers like a nerve, while the FOEW and TFBG-SPR sensors introduced in this part can observe the state of electrode interfaces in detail like dermatologists. While in theory, total reflection will occur when light is emitted from an optically dense medium to an optically thinner medium, both practical observations and theoretical developments increasingly demonstrate that leaked energy can be detected in a thin layer (approximately 100 nm) at the optically dense–thinner interface [67]. This phenomenon is referred to as an evanescent wave. By using this principle and combining it with an optical fiber sensor, a detailed “diagnosis” of the electrode surface can be achieved, as shown in Fig. 4a.

In a battery, pristine graphite has a relatively low free carrier concentration (∼10^−4^ free carriers/atom) at room temperature. As lithium ions intercalate and transfer/delocalize their 2*s* electron into the graphite π-band, the carrier density of the graphite layer adjacent to the lithium layer increases. This increase in the free carrier concentration causes alterations in the graphite’s electrical, chemical, thermal, magnetic and optical properties [68]. Through the interaction of the aforementioned FOEW sensor with graphite particles in a lithium-ion battery in multiple cycling, studies have found evidence indicating that the optical transmissivity of the evanescent wave is related to the lithium-ion concentration on the surface of the graphite particles [42, 69]. The optical intensity is associated with the degree of lithiation on the electrode surface, and the lithium ion concentration becomes the main factor affecting the transmissivity. As the battery cycling continues, aging inevitably occurs. In this case, the loss of recyclable lithium results in a reduction of the lithium content in the active material, and this degradation effect is reflected in the light spectral characteristics (Fig. 4b). In the previous section, a TFBG can “see” the electrolyte turbidity process; however, changes in electrolyte turbidity or salt concentration will not affect the observation of transmission by an FOEW sensor, meaning that the salt concentration in the electrolyte will not affect the evanescent wave signal [41]. Thus, by eliminating the related influencing factors through this phenomenon, the measured quantity is only related to the lithium ion concentration. Considering the short penetration depth of evanescent waves, the response of an FOEW sensor is likely to be affected by the surface conditions of electrode particles near the sensing area [41].

In the previous analysis, we can see that the FOEW signal shows sensitivity to the lithium concentration on the surface of graphite particles. By monitoring the evolution of the optical transmissivity, the relationship between the transmission amplitude and available capacity can be obtained, which facilitates monitoring the battery’s State of Health (SOH) [42]. When the capacity of the lithium-ion battery declines after long cycling due to the decrease in the lithium content of recyclable graphite, the FOEW transmission amplitude decreases. The transmission amplitude of an FOEW sensor has been successfully correlated with the SOH of the battery [70].

Like an FOEW sensor in sensing characteristics (electrode surface monitoring), an SPR sensor uses the strong electromagnetic energy localization effect in the layer adjacent to a metal surface by applying a thin metal coating to the optical fiber surface. Through the stimulation and detection of optical signals, surface plasmon waves can offer heightened sensitivity of chemical changes in the battery [45, 72], as shown in Fig. 4c, d.

Using the TFBG mentioned in the above section, the surface of the TFBG is coated with a metal layer so that the SPR sensor has both the TFBG multimode characteristics and SPR sensitivity. In a TFBG, the core mode is completely insensitive to electrochemical changes. Therefore, the spectrum of the core mode can be referenced in terms of the power and wavelength to eliminate the impact of any system instability (it acts as an in-situ thermometer, monitoring changes in wavelength, for example, intensity change or the wavelength of the core mode) [45]. When a gold-plated TFBG sensor is closely attached to an electrode surface, the change in the charge density and ion distribution around the electrode can be directly monitored through the change in the SPR signal [45], for example the SPR power evolution. So, it’s evident that the differential curve of the SPR power corresponds to the real-time change rate of the ion concentration. The two characteristic peaks in the discharge stage correspond to the maximum change rate of ions. And the mapping of the differential SPR power with the voltage corresponds to the change rate of the ion concentration under different potentials [73].

The previous section introduced that the use of TFBG cladding mode resonance peak can achieve detection of changes in the electrolyte environment state by monitoring changes in the refractive index of the electrolyte around the fiber. However, the use of TFBG higher-order cladding mode and mutual excitation of SPR with the metal film material on the grating surface can achieve higher sensitivity in monitoring changes in the internal environment of the battery. Compared to bare TFBG, the internal environment monitoring of the battery under the TFBG-SPR mechanism can achieve higher sensitivity in monitoring, and it has smaller temperature cross-sensitivity, which can greatly reduce the impact of temperature factors on measurement and simplify the measurement device.

The decomposition of SEI [74], the occurrence of graphite delamination effect [74], and the generation of combustible gases [75] can all impact the stability of the battery’s internal environment. The entry of related substances into the electrolyte, the reaction with the electrolyte, and even the consumption/depletion of the electrolyte can affect both the liquid refractive index and the interface refractive index of the battery’s internal environment. Evidence of battery degradation process can be found through historical comparison in optical fiber spectroscopy.

Based on mechanism analysis, both the FOEW sensor and SPR sensor need to be closely attached to the electrode surface due to the short penetration distance of energy. In this case, due to the blocking of lithium ion diffusion during the battery lithiation process, lithium will be enriched around the optical fiber [44, 70], leading to local electrode variation and even lithium plating. Now, it is doubtful whether the monitored electrode characteristics can represent the overall situation. A more serious issue is that if the enriched lithium pierces the separator [45], it will trigger thermal runaway, endangering the battery safety. Embedded sensors pose a ‘double-edged sword’ effect-monitoring safety and threatening it. Researchers must address this challenge.

For FOEW sensors, stable observations are typically achieved using silicon dioxide material. In a study exploring the aging performance of optical fibers in more severe environments, traditional silicon dioxide was replaced with high-polymer plastic optical fibers. Accelerated aging tests were conducted, and the experiments showed that, under acidic corrosive conditions, the optical fibers exhibited no abnormalities after cycling for 1000 h at 50 °C and 2000 h at 60 °C [76]. The fiber’s attenuation remained consistent. Considering this, we conducted a reasonable extrapolation, anticipating that FOEWS with silicon dioxide material could maintain stable operation in corrosive environments. TFBG-SPR sensors, like traditional telecommunications-grade optical fibers, also use mature silicon dioxide material and a gold metal coating. A study clearly indicates that, through proper encapsulation, as long as the geometric deformation of the optical fiber does not exceed its limit state, TFBG-SPR sensors can maintain sufficient stability and correct output in battery environments to achieve accurate parameter observations [45].

### Rayleigh Scattering Optical Fiber Sensors: Global Thermal–Mechanical Monitoring

**Fig. 5:**
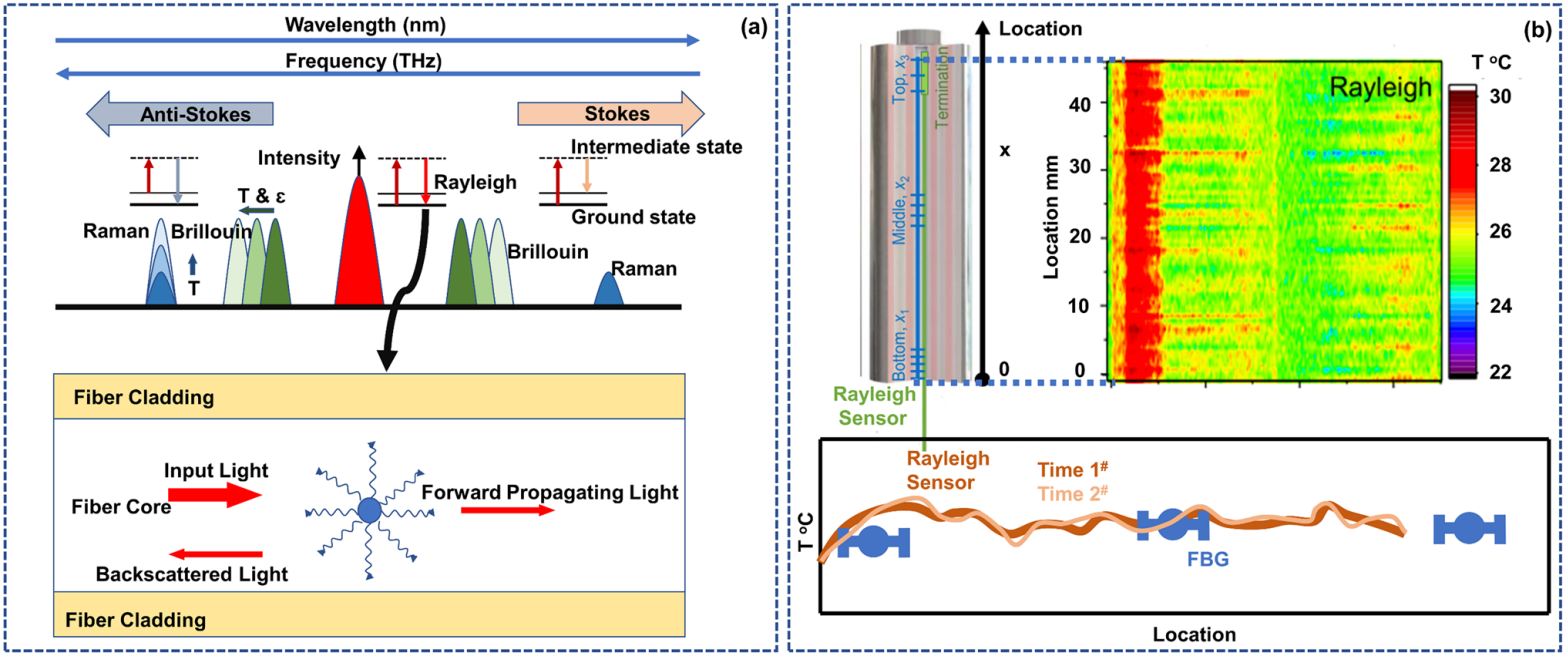
Distributed optical fiber sensing system based on the Rayleigh scattering principle. **a** Measurement mechanisms for different distributed optical fibers. When the particle size is much smaller than the wavelength of the incident light (less than one tenth of the wavelength), the scattered light intensities in each direction are different, and the intensity is inversely proportional to the fourth power of the wavelength of the incident light. This phenomenon is called Rayleigh scattering. Reproduced with permission from Ref. [39]. Copyright 2021 IOP Publishing. Reproduced with permission from Ref. [51]. Copyright 2019 AIP Publishing. **b** Comparison between distributed temperature measurement based on Rayleigh scattering effect and FBG quasi-distributed temperature measurement. The spectrum of temperature measurement based on Rayleigh scattering effect in time and space is more intuitive than that of FBG measurement, and the identification of temperature hot spots is more accurate. Reproduced with permission from Ref. [39]. Copyright 2021 IOP Publishing

The sensors introduced in this section based on the Rayleigh scattering principle are like physical examination doctors, capable of monitoring the overall mechanical and thermal health status of the battery. The sensors introduced above, whether structural FBG, TFBG sensors or functional SPR and FOEW sensors based on optical principles, have an inherent disadvantage; that is, they can only measure the battery status at one single point at the functional location. If more functional nodes are added, then these sensors can theoretically measure data at multiple points in the battery to form a quasi-distributed measurement structure but still cannot globally monitor the status of the battery (along the optical fiber path). Correspondingly, distributed optical fiber sensors using Rayleigh scattering, Raman scattering or Brillouin scattering can measure the full path range along the optical fiber distribution. Raman scattering is primarily sensitive to temperature, making it challenging for multiparameter measurements in batteries with structural changes [77–79]. The self-emitted Brillouin scattering has a weak light intensity, with restricted resolution and response time for related sensors [51, 80]. Due to the inherent multisource parameter sensing capability and high spatial resolution [46], distributed sensors based on Rayleigh scattering have been increasingly studied and have become mature and practical, as shown in Fig. 5.

The distributed sensor based on the Rayleigh scattering principle can be used to conduct real-time online measurements of the global state of the battery with very high spatial resolution. An experiment on the 18650-type lithium-ion battery showed that this kind of distributed measurement method can enable temperature and strain imaging of the battery with millimeter-level spatial resolution [39, 81, 82]. The high-precision measurement of global physical parameter, including distributed temperature, coupled with corresponding technical algorithms, can provide a more detailed analysis of the battery's operational status [83]. The use of distributed sensors based on the Rayleigh scattering principle to achieve high-precision estimation of battery core state parameters is more convenient when reducing equipment demand after calibrating relevant internal and external battery state equations in advance [84]. Distributed sensing can provide increasing amounts of abundant battery state information so that a more complete grasp of the battery can be obtained. Theoretically, in general battery applications, distributed sensors can be segmented and only collect parameters at fixed positions, making them similar to FBGs, which can reduce the amount of data. When abnormal conditions are detected, the fault location can be identified in this application through dynamic adjustment of the monitoring points (in this case, the sensor is similar to an FBG with an adjustable grid spacing).

One of the advantages of optical fiber sensors based on Rayleigh scattering, compared to several sensors mentioned earlier, is their capability for distributed measurements, a characteristic emphasized repeatedly in the paper. An essential requirement for achieving high-resolution monitoring of battery states along the entire distribution path of optical fibers is the uniformity and stability of the fibers at various positions. Any variation in the properties of the fiber at a specific location can lead to errors in the corresponding sensing. In this regard, Yu et al. [85] implanted Rayleigh scattering optical fibers into the interior of 18,650 batteries. After over 60 cycles, they found that the impact of the optical fibers on the batteries was nearly negligible. Furthermore, through continuous monitoring for 20 h, two different Rayleigh scattering optical fibers demonstrated highly reproducible temperature monitoring in the batteries. These findings demonstrate that distributed fibers within the battery can ensure continuous and stable operation for extended periods, ensuring the long-term performance stability of embedded sensors.

The characteristics and possible application scenarios of different optical fiber sensors are shown in Table 1, which can guide the selection of relevant optical fiber sensors in practical use.

**Table 1:** Characteristics and application scenarios of different types of sensors

Working mechanism	Sensor	Characteristic	Application scenario	Note	Refs.
Optical structural component	FBG	Simple structure Good economy Easy identification of measurement parameters High linearity	Macro-temperature and strain measurement	Installed inside or outside the battery	[36, 62]
	TFBG	No cross-sensitivity of temperature to refractive index measurement	Measurement of composite refractive index of electrolyte	Embedded installation inside the battery	[49]
Optical functional Component	FOEW	The change of electrolyte salt concentration is not related to the observation of transmissivity index	Transmission measurement of electrode-optical fiber interface	Close to the electrode surface	[71]
	TFBG-SPR	Integrated with TFBG can realize multifunctional applications	Measurement of charge density and ion distribution concentration on electrode surface	Close to the electrode surface	[73]
	Rayleigh Scattering sensor	Distributed measurement	Distributed observation of cell temperature and strain on optical fiber path	Installed inside or outside the battery	[83, 85]

Now, with the feasible implementation of the technology, advanced optical fiber sensors can monitor and diagnose battery strain, temperature, electrolyte and electrode degradation. The distributed sensors can be used in daily applications to achieve comprehensive physical examination of the battery “physical state” and “nidus” identification. With its small size, high stability and environmental adaptability, the advanced optical fiber sensor will be of excellent help in realizing battery intelligence in the future.

## Embedded Optical Fiber Sensors Help Strengthen Battery Safety

Sensing the changes in the basic properties (stress, strain, and temperature) of a battery is not the key problem for the application of embedded sensors in the battery; instead, the “mission” of embedded optical fiber sensors is to determine the phase change process, electrochemical behavior, interface dynamic response characteristics and safety evolution characteristics of the key materials inside the battery. Thus, optical fiber sensors can provide insight into the battery core reaction state to help enhance safety. In this section, in-situ real-time online monitoring of the battery phase change behavior, electrode and interface dynamics and evolution of battery safety characteristics using advanced embedded optical fiber sensors is reviewed.

### Early Warning of Battery Health State Enabled by Mechanical-Thermal Signals of Optical Fiber Sensors

According to the working principle of the battery, during the charging and discharging process, lithium ions are periodically inserted into and removed from the positive and negative electrodes through the electrolyte. In this process, there will be redox reactions near the cathode and anode interface [86, 87]. During this process, an SEI will be generated on the anode surface. At the same time, the material lattice is “filled” by and “stripped” of lithium, leading to expansion and contraction of the battery [7, 88, 89]. With the redox reaction, the battery releases heat, and the temperature synchronously rises. As the battery cycling continues, thickening of the SEI and the formation of gas due to the liquid electrolyte reaction will lead to an increase in the battery volume [90], and the abnormal side reactions will lead to an abnormal temperature rise in the battery [91]. Furthermore, the transformations in optical fiber signals caused by electrolyte aging, floating of dead lithium, and various physicochemical changes in batteries have been thoroughly discussed in the preceding parts.

From the above observations, we can see that, firstly, during the battery’s service life, monitoring changes in the battery’s volume can detect a decline in the health status before electrical and thermal signals. Specifically, as lithium ions cycle and the breakage-reformation of the solid electrolyte interface (SEI) occur, active lithium is continuously consumed. If judged solely from a capacity perspective, there is a long period of linear capacity decline before a “dive” in the capacity of lithium-ion batteries. However, from a mechanical perspective, noticeable accelerated aging behavior of battery expansion can be observed several days or even weeks before the capacity dive, providing an early indication of the decline in the battery’s health status. Furthermore, by utilizing optical fiber measurements of changes in battery expansion behavior and employing simple binary thresholds, we can detect the occurrence of phenomena such as lithium plating very early on. This helps prevent accidents and disasters caused by lithium plating, providing an early warning for the battery’s deteriorating health status. In addition, based on the mechanical characteristics of optical fibers, through simple technical operations, it is currently possible to observe the battery expansion caused by the thickening of SEI, lithium plating, and gas generation. The expansion rates and characteristics of these three phenomena have distinct differences. Through further research, if decoupled observations of the three main expansion behaviors can be achieved, the mechanical monitoring of battery health will be further refined, potentially allowing for a more detailed differentiation of the evolution of battery health. Then, temperature is highly sensitive for batteries, and optical fiber sensors embedded in each cell can provide detailed temperature monitoring. This offers a more accurate and comprehensive data foundation for the thermal management system, helping to ensure uniform temperatures within the battery pack and contributing to extending the battery’s lifespan. Regarding the health of the electrolyte, there is a significant difference in the spectral signals between healthy electrolyte and electrolyte with suspended impurities. With further research, if we can extract the spectral signal characteristics corresponding to different impurities and concentrations, we can conveniently detect early signs of events that may harm battery health and safety.

Visible, the sensitivity of advanced optical fiber sensors to temperature-strain and the refractive index of the liquid environment enables simultaneous observation of the macro- and micro-properties of the battery and prediction of the aging state of the battery [85, 92]. Therefore, based on macroscopic changes in the battery’s physicochemical characteristics, such as temperature and stress (strain), as well as microscopic changes, such as alterations in refractive index, the battery can perceive the early evolution of its own health state.

Because lithium will be deposited on the surface of the metal sensor, incomplete insulation treatment and sensor metal burrs may cause short circuits in the battery. Some studies have implanted thin-film metal sensors into batteries. Although these sensors with extremely small out-of-plane dimensions seem well-suited for monitoring the internal electrothermal characteristics of batteries, it is easily understood that these sensors generally have larger in-plane dimensions. This planar sensor design often hinders ion transport at its location, leading to increased polarization and impacting battery performance. With the increasing demand for fine management of batteries, the conventional approach of monitoring module voltage and surface temperature is no longer suitable for existing battery systems. As research advances, more conclusions indicate that when voltage sensors or thermocouples detect abnormal voltage signals or temperature rise in the module, the internal temperature of the battery may have already surpassed irreversible thermal runaway characteristic temperatures. At this point, such delayed monitoring may not be conducive to early warnings for battery safety. It is also essential to emphasize that with a substantial increase in the number of batteries in energy storage systems, achieving fine control with traditional electro-thermal sensors may require installing sensors for each individual battery. Faced with systems composed of thousands of batteries, this approach leads to an exponential increase in the complexity of signal reception and processing, demanding extensive software and hardware requirements. Traditional battery sensors are increasingly unable to meet the demands of fine battery management and technological development.

The material properties and geometric characteristics of optical fiber sensors enable them to adapt to the complex and harsh environments within batteries. Embedded optical fiber sensors can promptly detect anomalies in the battery that are imperceptible externally. Additionally, the multiplexing capability of optical fiber sensors makes them highly suitable for scenarios with a large number of batteries. Taking the example of a standard Fiber Bragg Grating (FBG) sensor, current technology allows the inscription of 30 gratings on one single FBG. Consequently, one optical fiber cable can provide real-time monitoring for 30 batteries, significantly reducing the hardware interface requirements to one-thirtieth of the original. With further understanding of the time domain and frequency domain characteristics of optical fiber, some companies (e.g., Changfei in Wuhan) can inscribe tens of thousands of gratings on a single fiber. In theory, for an energy storage station comprising tens of thousands of batteries, a single fiber optic sensor could achieve the effects that would traditionally require tens of thousands of regular sensors. This is highly advantageous for fine battery management.

**Fig. 6:**
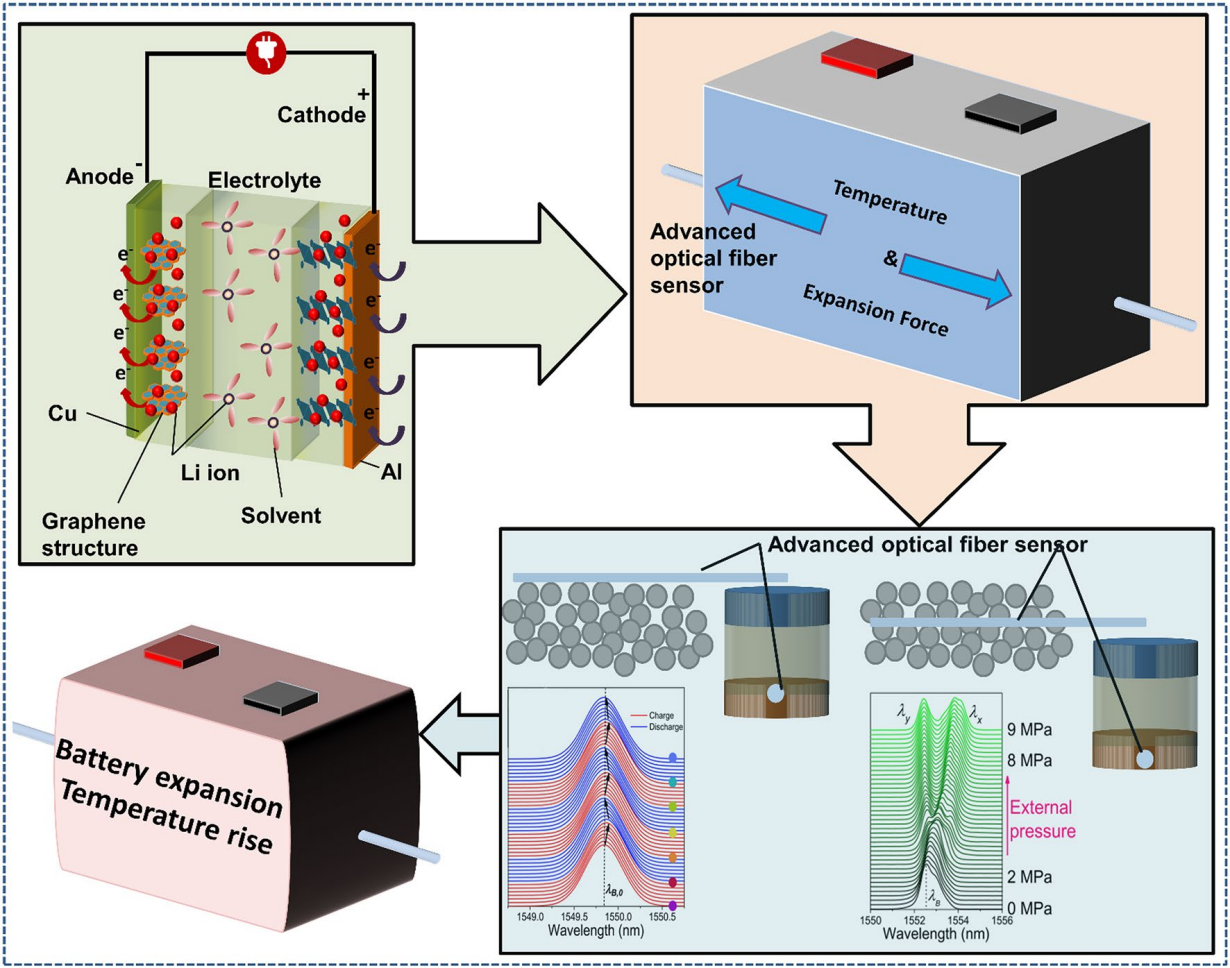
Observation of the “breathing” effect of the battery by an advanced optical fiber sensor. The fiber has different wavelength response behaviors at different interface positions, and the bidirectional stress inside the electrode causes splitting of the fiber wave peak, indicating anisotropy of the electrode active material. Reproduced with permission from Ref. [99]. Copyright 2022 Springer Nature

Thanks to its intrinsic safety, a silica-based optical fiber sensor has a unique advantage in gaining insight into the internal state of the battery. By implanting the functional optical fiber into the electrode interface and the electrode active material, different characteristics can be observed. As shown in Fig. 6, due to the axial and transverse alternating stress effect within the active material, the optical fiber embedded in the material exhibits a peak splitting characteristic that differs from the optical fiber at the material interface [86]. The occurrence of this phenomenon is closely related to the embedding of lithium and the resulting multidirectional expansion of the active material, insights that are inaccessible to external surface sensors of the battery. In addition, remarkable findings have been discovered through the use of optical fiber sensors. The diffusion of lithium in the electrode is not uniform. The interaction between fast and slow lithium-ion diffusion processes leads to uneven insertion of lithium ions [93, 94]. The natural observation advantage facilitates an understanding of the charge and material transmission characteristics, as well as elucidation of the relationship between the changes in the electrode structure and thermal-mechanical stress at the electrode–electrolyte interface [95]. Beyond conventional battery state estimation, an apparent application value lies in enhancing the safe usage of high-performance batteries [96, 97]. The battery mechanical, electrical and thermal characteristics can be adjusted based on the information of optical fiber sensors so that the battery always has the best performance and safety. In general, the contradiction between “fast” and “stable” that is never easy to tune in the field of fast charging (high power discharging) applications can be overcome [98].

### Electrochemical Reaction State Reflected by Spectral Signals of Optical Fiber Sensors

In the previous section, the importance of monitoring the constitutive properties of the battery, such as the temperature, stress and strain, was discussed. If this basic information about the battery is unclear, then mastery of the key factors that affect the battery life and service performance, such as solid–solid and solid–liquid interface properties and dynamic characteristics, will be lost [36]. Some research teams have proven that the chemical information contained in the optical sensing signal can help in understanding the characteristics of battery parasitic reactions and interface growth dynamics [36]. Embedded optical fiber sensors are similar to “non-invasive inspection”; they can clarify the electrochemical reaction process inside the battery.

**Fig. 7:**
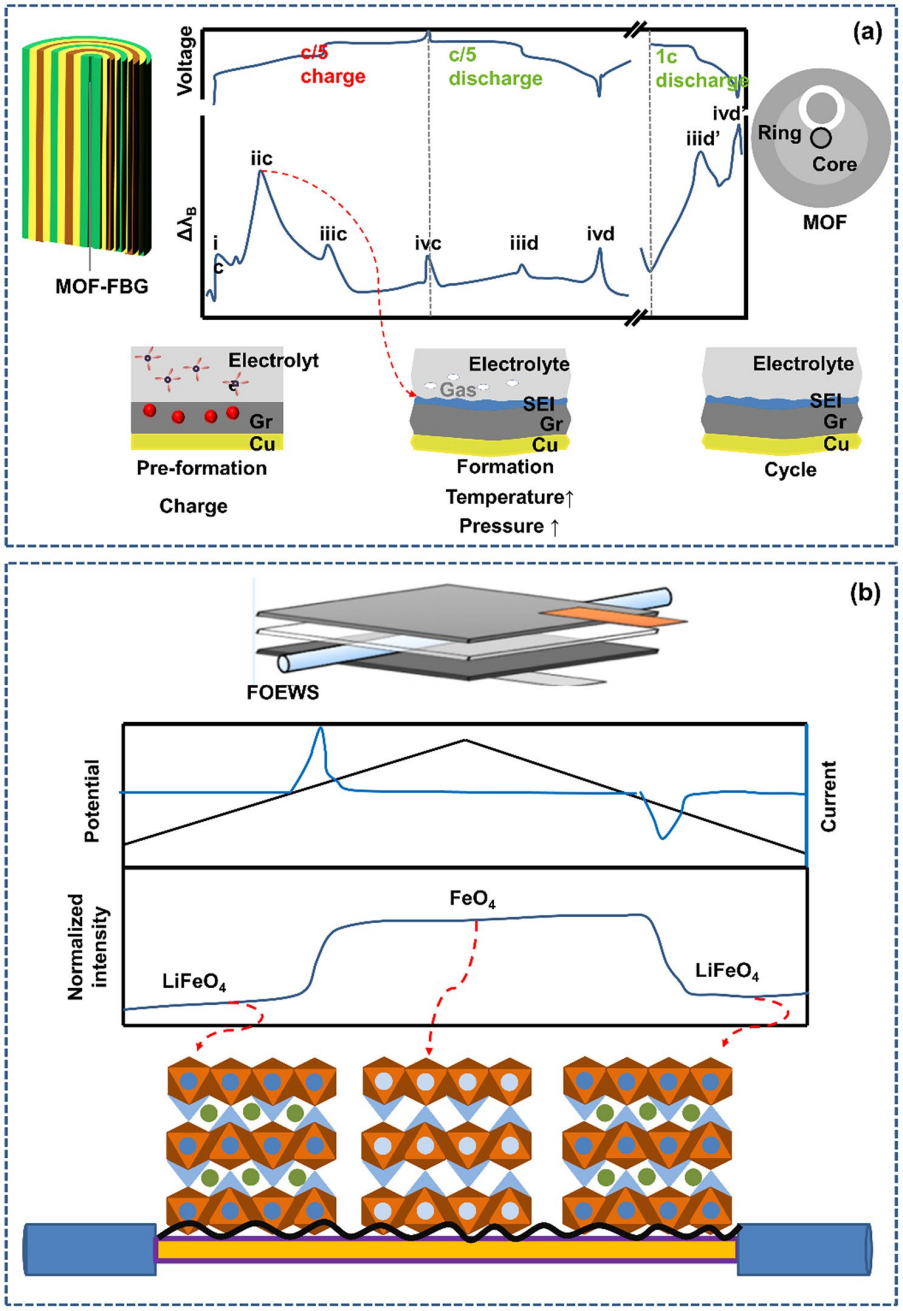
New understanding of the electrochemical reaction through advanced optical fiber sensors. **a** An improved microstructure optical fiber was used to understand the electrochemical reaction state of the battery by observing and calculating the heat release, chemical reaction at the electrode–electrolyte interface and reactivity of electrolyte additives. Reproduced with permission from Ref. [36]. Copyright 2020 Spring Nature. **b** Understanding the relationship between the redox of Fe in an LFP-based battery and the strength of the optical fiber output, and understanding the material reaction process. Reproduced with permission from Ref. [41]. Copyright 2021American Chemical Society

The results of monitoring the temperature rise and pressure change characteristics inside a battery utilizing an optical fiber sensor with a modified structure are shown in Fig. 7a. The appearance of the peak-iic temperature rise is accompanied by an increase in the pressure, this indicates the generation of gas. Notably, this phenomenon is absent in the subsequent cycle, suggesting that this effect is linked to the formation of an SEI [36]. In addition, the unique optical sensing method can be used to adjust the interface chemistry through heat release, chemical reaction at the electrode–electrolyte interface, and reaction activity of electrolyte additives. This approach enables a comprehensive understanding and optimization of the battery material ratio to attain superior performance [54].

The use of advanced optical fiber sensors can bring a new understanding of and thinking about the electrochemical reactions. Taking a lithium iron phosphate (LFP) battery as an example, an intuitive and robust correlation can be established between the optical signal sensed by an optical fiber and the concentration of lithium or even the redox reaction of battery elements [42]. As shown in Fig. 7b, when the LFP is oxidized, the signal strength increases. When most of the LFP is oxidized, the strength remains unchanged, and then, the strength decreases when the current drops below zero. At this time, FePO_4_ is reduced to LiFePO_4_. After a complete redox cycle, the signal strength recovers to the same level [41]. More detailed analysis revealed that because the charge is controlled by the potential, the current generated is related to the amount of iron oxidized/reduced [43]. When the LFP is fully charged or discharged, the current drops to almost zero, which means that the optical response alternates between two levels of values and remains constant. Starting from the charged state, when the potential becomes sufficient to oxidize increasing amounts of Fe^2+^, the current increases, and lithium ions are extracted from the olivine structure. After a period of time, the current becomes limited due to mass transfer. When all Fe^2+^ is completely oxidized, the current gradually drops to almost zero. According to this phenomenon, chemically lithiated Li_0.6_FePO_4_ shows a higher absorption intensity than delithiated FePO_4_, which leads to a high light intensity in the charged state and a low light intensity in the discharged state [43].

The above analysis clearly indicates that the changes in relevant spectral signals, combined with electrochemical theory can perfectly explain the electrochemical reaction process inside the battery. From this, it can be seen that when the monitored active material undergoes aging, its reaction intensity will change. By comparing historical changes in spectral data, it is very easy to obtain the cause of the failure. To put it further, it can at least eliminate certain probability events and help researchers carry out research in the correct direction. In normal use, an advanced optical fiber sensor is very important for obtaining a deep understanding of the redox reaction and accompanying battery processes, which can help in adjusting the working conditions of the battery in time, improving the battery’s working conditions, and maximizing the service life of the battery.

**Fig. 8:**
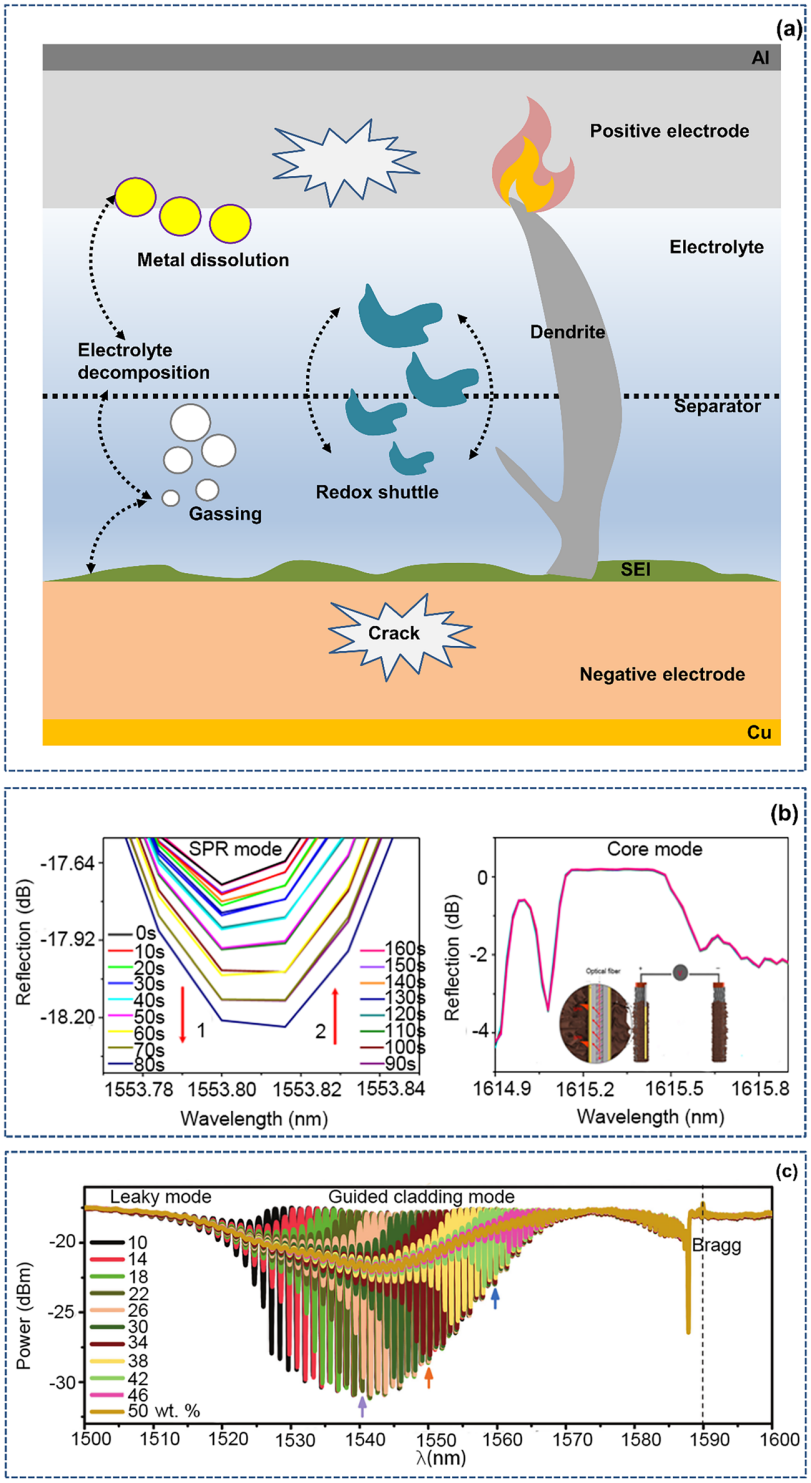
An advanced optical fiber sensor provides insight into the internal environment of an electrolyte. **a** Factors that cause deterioration of the internal environment of the battery electrolyte during the entire life. Reproduced with permission from Ref. [52]. Copyright 2022 Springer Nature. **b** Effect of different electrolyte concentrations on the light intensity in the SPR mode and core mode. Reproduced with permission from Ref. [45]. Copyright 2018 Springer Nature. **c** Overall effect of the salt concentration on the light intensity. Reproduced with permission from Ref. [49]. Copyright 2021 Royal Society of Chemistry

In the process of long-term cycling, a problem that cannot be ignored is the occurrence of detrimental side effects, as shown in Fig. 8a, including physical and chemical effects. Electrode cracking causes damage to the integrity of the battery structure, which may lead to shedding of active materials. Taking metal dissolution as an example, on the one hand, the dissolved metal will change the electrolyte environment; on the other hand, it will hinder the insertion of active lithium, resulting in attenuation of the battery life and capacity [100]. Moreover, shuttling of redox contaminants in the electrolyte and serious lithium plating [52] greatly harm the battery performance and safety. An optical fiber sensor can intuitively perceive the changes in the electrolyte “internal environment” (refractive index, transmissivity) [89] through displacement of the peak signal and changes in the optical signal energy [52], as shown in Fig. 8b, c, and then the possible battery attenuation and performance degradation of electrodes, interfaces, and materials can be deduced.

### Large Margin Early Warning of Battery Thermal Runaway Through Optical Fiber Sensors Signals

A troublesome problem for battery safety is the identification and early warning of thermal runaway. Common battery thermal runaway safety warning methods are limited by the sampling channel, data volume, and sensor technology and can only measure the voltage and surface temperature at several nodes at the module level [7, 101], detect the gas in a ventilation channel, or monitor safety using the battery’s impedance characteristics [102]. These technologies have some shortcomings, limited by the sampling principle and sampling location (usually sampling is conducted outside the battery), when the system detects abnormal signals, the battery may already be in an irreversible failure state, or the battery structural integrity may have already been fatally damaged. This kind of “hindsight” safety supervision and early warning is obviously not suitable for high-margin pre-warning of battery issues [103].

An optical fiber sensor, as described in the previous part, can perform real-time online monitoring of the internal environment of the battery. With the characteristics of being small, stable and accurate, it can be used to achieve embedded and advanced battery safety early warning, and catastrophic failures and extreme abuse can be instantly identified [52, 104]. In this way, the embedded optical fiber sensor endows the battery with “pain sensing nerves”, allowing the battery to perceive danger earlier and more reliably.

**Fig. 9:**
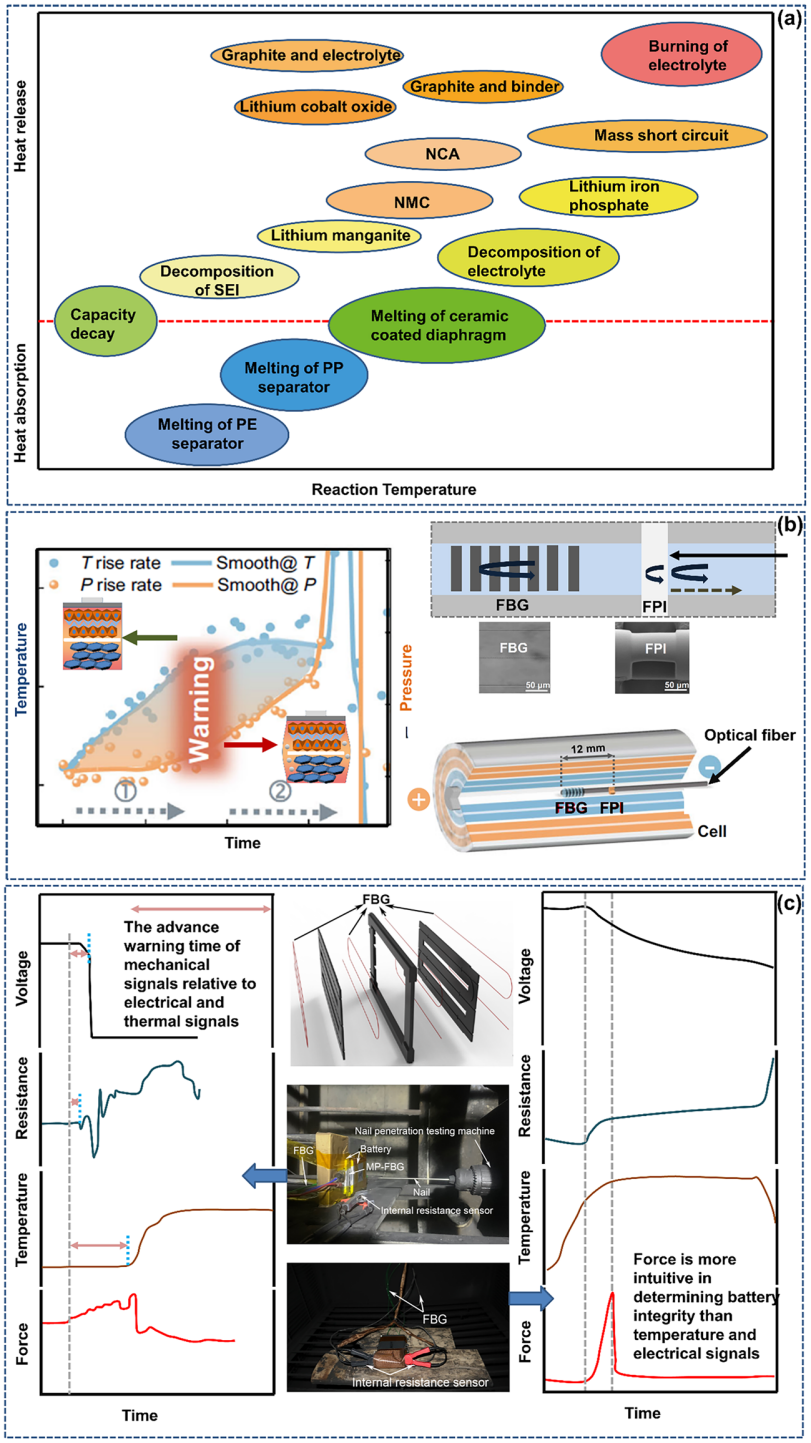
An advanced optical fiber sensor exhibits a timely response to dangerous battery thermal runaway. **a** Reaction sequence of components and materials in battery thermal runaway. **b** New multifunctional optical fiber sensor for monitoring the thermal runaway of commercial lithium-ion batteries. Reproduced with permission from Ref. [62]. Copyright 2022 Spring Nature. **c** SLIB equipped with an FBG sensor gives an early warning of extreme abuse conditions. Reproduced with permission from Ref. [114]. Copyright 2022 Elsevier

When the battery is continuously subjected to abuse conditions, whether mechanical abuse [105, 106], electrical abuse [107] or thermal abuse [108], it will eventually evolve into a state of thermal runaway. As shown in Fig. 9a, thermal runaway typically includes the following processes: First, the SEI decomposes, then the anode collapses, and the embedded lithium reacts with the electrolyte. The positive active substance decomposes with the electrolyte lithium salt, the electrolyte solvent is oxidized, and the embedded lithium reacts with the adhesive. The separator is melted or broken, and the positive and negative electrodes are short circuited [109, 110]. During this period, the battery temperature changes, and a large amount of gas is released [111, 112].

Based on the above thermal runaway mechanism and sensing mechanism, advanced optical fiber sensors can be “symptomatically” used to achieve prewarning of thermal runaway. The most macroscopic approach is that when a battery has an abnormal temperature rise or swells, the abnormal state beyond the threshold can be easily detected by using the temperature-strain sensitivity characteristics of an optical fiber. When the optical fiber is embedded in the battery, if the electrode is damaged due to aging or internal short circuit, then the optical transmittance at the interface will abnormally change. When the scattered particles fall off or the electrolyte ages or deteriorates, the refractive index of these components will accordingly change. The optical fiber sensor can still easily give abnormal state warning information at the early stage of battery attenuation. Compared with simple electrical and thermal warning signals (studies have noted that the voltage signal in large modules cannot be used as a reliable warning signal for thermal runaway [113]), the change in this substantive optical signal is not only highly sensitive but also has a great safety margin. The joint team used a new multifunctional optic fiber sensor to operando monitor the thermal runaway behavior of lithium-ion batteries. The key timing of thermal runaway warning was summarized through the parallelogram formed by the temperature-pressure data transition point measured by the optical fiber sensor (Fig. 9b) [62]. Li et al. [114] successfully realized reliable early warning of the battery safety status under extreme abuse conditions at the cell level using a newly designed optical fiber structure, as shown in Fig. 9c. Reghavan et al. [115] built optical fiber sensors in lithium-ion batteries that receive input light and provide output signal light according to the change in the amount of free or dissolved gas in the battery. These studies are important practical steps toward using embedded optical fibers to carry out thermal runaway warning for batteries. More practically, at the module level, if the optical fiber is encapsulated in a thin Teflon tube or tape, then this technology can enable users to measure a span of 50 m with a spatial resolution of less than 1 mm, which is beneficial for improving the battery safety performance and reducing the data volume and channel number [116].

Optical fiber sensors provide early warning of thermal runaway at the mechanical level, and enhance battery safety from an engineering strategy perspective. On this basis, more effective battery body design is supplemented, such as the application of improved SEI [117], new high safety additives [118], high safety separators [119, 120], high safety metallized plastic foils [121], and even in-situ polymerized solid-state batteries [122] and other new materials, components, and systems. This will greatly enhance the safety warning ability of batteries in response to mechanical, electrical and thermal abuse and improve safety tolerance [123, 124].

It is worth noting that when the battery is facing thermal runaway, its high temperatures of hundreds or even thousands of degrees Celsius [123] will pose a great threat to the stable operation of optical fiber sensors. Conventional ultraviolet etched gratings may fail in this environment, while femtosecond written optical fiber gratings can adapt to this high-temperature environment for continuous operation. In more cases, thermal runaway of batteries will be accompanied by violent explosions, and strong impacts can easily destroy fragile optical fiber bodies, such as fiber breakage, which also poses a challenge to the accuracy and normal operation of optical fibers. Currently, high-strength optical fibers that can adapt to such strong impacts are rare. In the future, the universal high-temperature and high-strength optical fiber that can adapt to normal and abnormal conditions during the use of batteries or energy storage devices, as well as during pre-disaster, disaster, and post-disaster environments, will be one of the important links in the development stage of battery-embedded sensors.

The development of lithium-ion battery technology should not only meet the requirements of service performance (energy performance, power performance, cycling performance) but also fully ensure safety. Since the first commercial lithium-ion battery was developed in the last century, a variety of these potential battery materials have emerged. However, potential battery materials cannot achieve global optimization in terms of safety and usability. Almost no material is completely safe and highly suitable for use. The existing battery materials can no longer meet the needs of society. Moreover, our understanding of the electrochemical reactions inside the battery is still insufficient, and battery safety accidents frequently occur, which not only restricts the promotion of lithium-ion batteries but also aggravates social concerns. More importantly, the further development of battery technology will be greatly limited. With advanced optical fiber sensors, we can deeply understand the core reactions and the internal state of the battery, and this can better guide the development of technology toward the correct path.

The domain size of different optical fiber sensors can cover from the micro-element level to the macro-battery level, up to the module and even system level. The unique usage characteristics and achievable functions of different sensors are shown in Table 2, which can serve as a basis for selecting future optical fiber sensors in studying battery performance and status.

**Table 2:** Summary of the usage effects that different fiber optic sensors can achieve

Domain feature	Sensor	Monitoring Process	Principle	Function	Refs.
Element	FOEW	Observing the oxidation–reduction of elements	Change in light signal intensity	Understanding the redox process of elements and its accompanying processes	[41]
Interface	SPR	Observing the particle concentration at the interface	Changes in light intensity of SPR mode	Understanding interface electrical properties	[45]
Domain	TFBG	Observing the turbidity level of electrolyte	Changes in light intensity of guided cladding mode	Understanding the evolution of electrolyte domain performance	[49]
Macro: Point to Line *(Adding a time dimension can transform single measurement point information into line information)*	FBG	Observing the transverse and longitudinal bidirectional stress inside the battery; Observing oxidation–reduction temperature rise	FBG reflection peak splitting and displacement	Understanding the characteristics of charge and matter transport	[99]
Macro: Line to Surface *(Adding a time dimension can transform the information along the optical fiber into surface information)*	Rayleigh Scattering sensor	Observing global force and heat	Rayleigh scattering spectral shift	Understanding global force and thermal evolution	[85]

## Optical Fiber Sensors Drive Future Energy Storage Technology Progress

In the future, advanced energy storage technology will not rely solely on the technical route of lithium-ion batteries. The development of multiple high-performance energy storage systems will greatly strengthen the entire energy storage industry. Advanced optical fiber sensors have great adaptability due to their characteristics and good compatibility with various energy storage schemes. In the future, energy storage devices will never be simple “energy storage containers”. Energy storage devices such as batteries are bound to “grow nerves”, “form brains” “extend limbs” and become “living intelligent individuals” in the future “evolution” process. In this section, the application and development of advanced optical fiber sensors in various energy storage systems and the final application of smart batteries are prospected.

### Application of Advanced Optical Fiber Sensors in Various Energy Storage Systems

The above sections mainly summarize the application prospects of advanced optical fiber sensors in traditional liquid-based lithium-ion batteries. However, the current lithium-ion batteries have begun slowly fall behind the needs of technological development in terms of energy density, power density, resource abundance, safety, etc., and the existing technologies are gradually “squeezing out” the final potential of traditional lithium-ion batteries. Advanced and high-performance energy storage devices such as solid-state batteries [125], Li-S batteries [126], Li-Si batteries [127], Li-air batteries [128], Na-ion batteries [129], water-based batteries [130], fuel cells [131] and even high-performance supercapacitors [132, 133] with better performance and safety have been developed, and a large number of studies have affirmed the value of the future applications of these technologies.

**Fig. 10:**
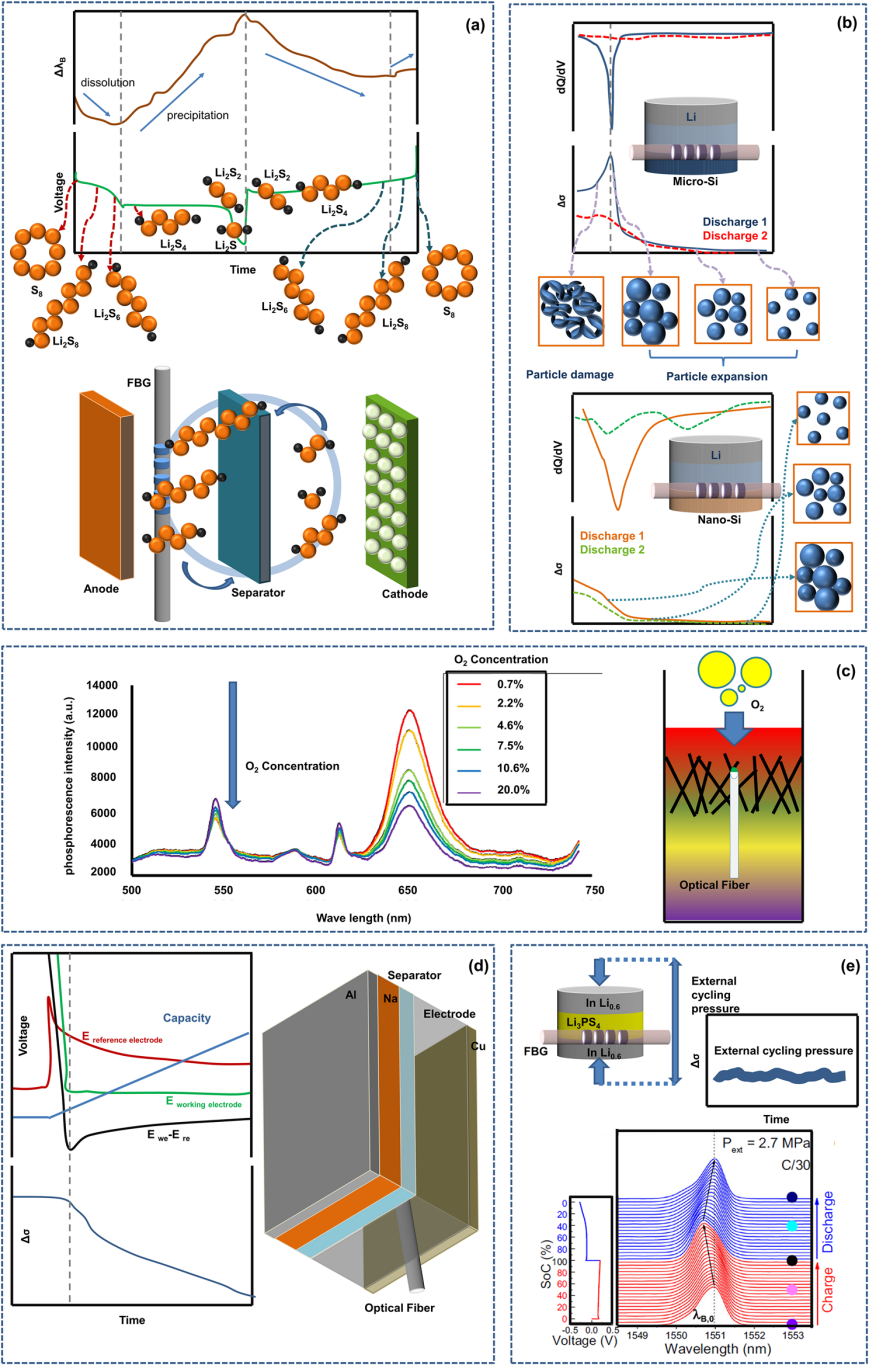
Advanced optical fiber sensors have great application prospects in various types of batteries. **a** In the Li–S battery, the optical fiber sensor identifies the key phase change process of the electrode. Reproduced with permission from Ref. [38]. Copyright 2022 Royal Society of Chemistry. **b** The optical fiber sensor monitors the entire process of the Si-based electrode from complete structure to particle damage. Reproduced with permission from Ref. [99]. Copyright 2022 Springer Nature. **c** The oxygen partial pressure in the Li-air battery affects the light intensity, which can be used to monitor the oxygen concentration. Reproduced with permission from Ref. [138]. Copyright 2019 The Japan Society of Mechanical Engineers. **d** The application of advanced optical fiber sensor in Na-ion battery. When the electric signal is applied, the optical signal drops rapidly, indicating the occurrence of sodium precipitation. Reproduced with permission from Ref. [71]. Copyright 2022 American Chemical Society. **e** FBG sensors at different locations found “breathing” and spectral peak splitting effects caused by solid-state batteries under pressure. Reproduced with permission from Ref. [99]. Copyright 2022 Springer Nature

As with traditional lithium-ion batteries, researchers are puzzled by some problems, such as the fuzzy internal reaction mechanisms of these promising energy storage devices, the unclear path of the entire-life-cycle performance evolution, and the opaque aging process. To overcome these obstacles, advanced optical fiber sensor technology will be a good tool to help researchers understand these issues. Li-S batteries which have higher theoretical capacity and resource abundance than traditional lithium-ion batteries are currently in the development stage of research, but their reaction mechanism is still unclear, which greatly hinders the progress of Li-S battery technology. Through built-in advanced optical fiber sensors, researchers have well explained the recognized phase change process occurring in the electrochemical reaction process under typical mechanisms, as shown in Fig. 10a. For example, the sulfur positive electrode undergoes severe and complex stress changes during the charging and discharging process under the solid–liquid–solid mechanism. At the beginning of discharge, the S cathode experiences stress release due to the volume contraction of long chain molecules. However, due to the formation of solid phase products, the stress increases monotonously until the end of the discharge. Later scanning electron microscopy also found that the density of S/C composite electrode was significantly higher than that at the beginning of the discharge, which confirmed that the stress of S cathode had increased due to the increase of volume during the discharge process. The battery stress is released during charging. The above findings provide a new perspective for understanding the working mechanism of Li-S batteries. These results provide guidance for the future development of Li-S batteries from the perspective of mechanical stress chemistry [38]. Since the change in molecular structure causes the change of cell stress, which is keenly perceived by the embedded optical fiber sensor. The method useful for Li-S batteries is also applicable to other batteries, for example, such as Li-Si battery. The development of Si negative electrode batteries with a higher theoretical capacity has achieved great success, but the volume expansion of more than 2x is a large “stubborn” problem that restricts battery lifespan [110, 134, 135]. This kind of electrode stress greatly affects the change in the chemical potential [136, 137] and prevent electrode lithiation. At present, stress analysis of the initial lithiation process can be realized by using an optical fiber sensor, as shown in Fig. 10b. Through the stress analysis of the electrode, it is found that the electrode pore filling, electrode thickening, and electrode particle pulverization correspond to the smooth stress increase, the steep stress ramp and the stress release, respectively. Therefore, the stress curve observed during the first lithification process can elucidate the particle integrity evolution sequence of Li-Si battery. Consequently, tracking of silicon particle damage can be realized [99]. Finally, the integration of advanced optical fiber sensors can also be achieved in Li-air battery with the highest energy density, which uses the influence of the oxygen partial pressure on the light intensity to monitor the oxygen concentration in the porous cathode, as shown in Fig. 10c, achieving the goal of low-cost application and safe use [138]. Apart from novel batteries related to lithium ions, sodium-ion batteries have also garnered more attention in recent years due to their exceptional cost-effectiveness. Optical fiber sensors have been used to guide the optimization research of the sodium-ion battery cycling performance and the optimization selection of electrolyte salts. Interphase chemistry is an important indicator to promote the battery to achieve practical application performance. Advanced embedded optical fiber sensors can be used to track the battery interface reaction and thermal state, thus determining the role of different additives in the formation process of SEI/CEI (cathode-electrolyte inter-phase). This offers an expedited pathway for the progress of battery materials [36, 54]. In addition, with optical fiber sensors, researchers have accurately characterized the sodium evolution behavior that affects the battery safety and even directly observed the sodium metal coating in situ, as shown in Fig. 10d [71].

The solid-state battery is the most sought-after intrinsically safe battery at present, but the observation of external mechanical stress during its cycling cannot be used to evaluate its phase change process, so some key processes cannot be analyzed. A research team found an intriguing peak splitting phenomenon using embedded optical fiber sensors, as shown in Fig. 10e, which is closely related to the anisotropic expansion behavior of the solid-state battery [99]. This further characterizes the stress state that is completely different in the transverse and longitudinal directions [99]. In another experiment, the mechanical sensor was placed outside the solid-state battery, but the stress was almost unchanged. This is mainly because an increase in stress on one electrode in the solid-state electrode corresponds to and is equal to the release of stress on another electrode, so that the whole system is in a mechanical balance state. The optical fiber sensor provides an observation ability at the material level due to its sensitivity to local stress changes, which enables monitoring of mechanical properties that is much better than monitoring of the average change of the entire equipment.

It can be seen that advanced fiber optic sensors not only have excellent application prospects in traditional lithium-ion batteries, but also are suitable for other batteries system, and have very bright application prospects in many energy storage systems that may be deployed on a large scale in the future.

The characteristic of electrochemical neutrality benefiting from optical fiber sensing can be used for most non-water-based environment batteries (Li/Na-ion battery, Li–S battery, Li–Si battery, solid-state battery, etc.) or water-based environment batteries (Zn–MnO_2_ battery) [52]. The excellent features of advanced optical fiber sensors are applicable to almost all known energy storage systems, which will be greatly beneficial for understanding the mechanisms of energy storage devices and the evolution in the entire life process.

In addition to the applications described earlier, optical fiber sensors are highly suitable for various existing and future battery systems at the research level. In the previous description, fiber optics, with their fine 100 μm diameter, are well-suited for applications in small-sized batteries, such as coin batteries, Swagelok batteries and so on. By perforating both sides of a coin battery, fiber optics can traverse the electrode surface for real-time observation of the most fundamental single-electrode half-cell or exploratory full-cell, including mechanical, thermal, and electrochemical properties. The flexibility of fiber optics allows for the selection of sensing positions at will within coin batteries. Under certain specific research conditions, optical fiber sensors can be easily placed in the regions with the highest electrochemical activity to achieve optimal experimental results. Using Swagelok battery, optical fiber sensors can detect more sensitive sensor signal responses than pouch batteries. Leveraging the structural advantages of Swagelok batteries, fibers can easily pass through the middle of the battery, maintaining excellent sealing and ensuring the normal operation of electrode. After embedding fibers in Swagelok batteries, any volume changes in the electrodes will trigger a fiber response, meeting the demand for electrode monitoring to the maximum extent. The presence of optical fiber sensors enables direct contact with the electrode surface for online monitoring of the observed elements. When combined with a special battery equipped with a reference electrode (three-electrode cell), optical fiber sensors can directly judge the behavior of metal ion plating like Li-ion plating or Na-ion plating (cross-confirmation of electrical and optical signals). In pouch batteries, fibers can be placed at different positions inside the battery to observe the differences in the electrode’s electrochemical response behavior. This allows for monitoring the non-uniform behavior of the battery. Additionally, by leveraging the distributed or quasi-distributed measurement characteristics of fiber optics, multiple sets of batteries can be connected on a single fiber, reducing hardware requirements and minimizing measurement errors caused by sensor inconsistencies. The most direct method of embedding an optical fiber sensor in cylindrical or hard-shell batteries is to create openings at the mandrel or the top of the steel shell, implant the fiber inside the battery, and then seal the openings with sealant. One significant advantage of optical fiber sensing within the battery is the ability to promptly perceive changes in the internal temperature of the battery, avoiding the delayed external temperature rise compared to the internal temperature rise due to the low radial thermal conductivity. Simultaneously, it avoids the lag effect caused by temperature-based early warning of thermal runaway. As mentioned earlier, the small size of fiber optics makes it very convenient to embed them inside the electrode. In the application scenarios of solid-state batteries, by threading the fiber optic through the active material, the optical fiber sensor unexpectedly discovered the expansion effect of the electrode that was not detected by the external pressure sensor. The optical fiber sensor provides the possibility to insightfully observe the localized stress changes in the electrode at the material level. Finally, for the next generation of electronic devices, they will be driven by miniature components tailored for wearable options, which still need to power the devices. One of the most innovative approaches currently is the design of wearable batteries with flexible and textile characteristics. In this regard, optical fiber sensors possess unparalleled features. Their slender dimensions allow them to flex freely with the wearable battery (avoiding sharp bends). They might even serve as a fixed matrix for wearable batteries, playing a crucial role in the health management, safety monitoring, and safety warnings of flexible batteries. The application value of optical fiber sensors in different battery research scenarios is summarized in Table 3.

**Table 3:** Application value of fiber optic sensors in different battery research

Battery type	Embedding position	Function	Refs.
Coin battery	Symmetrically embedded in the middle of the cell	Traverse the battery, can be installed at any position; especially suitable for embedding at the most active electrochemical location	[99, 139]
Swagelok battery	Symmetrically embedded in the middle of the electrode	Achieves higher detection accuracy than pouch batteries after embedding	[68, 70, 99]
Three-electrode battery	Between electrode sheets, near the reference electrode	Cross-confirmation of optical and electrical signals; excellent binding performance	[71]
Pouch battery	Between electrode sheets	Can be embedded at any position, enabling measurement of battery non-uniformity; features a one-to-many characteristic with strong scalability	[35, 41]
Cylindrical battery	In the mandrel	Directly embedded in the battery core, avoids delayed judgment of internal temperature due to low radial thermal conductivity	[36, 39, 49]
Hard shell battery	Between electrode sheets		[140]
Solid state battery	Inside the active material	Able to detect the expansion issues of solid-state electrodes that conventional external sensors cannot detect	[99]
Wearable batter	Can be used as a battery matrix; can also be wound around the battery	Flexible mechanical properties and minimal radial size, can flex freely within flexible batteries, can be used as the base for supporting batteries	–

### Prospects of New Type Optical Fiber Sensors in Energy Storage Systems

Advanced optical fiber sensors such as FBG, TFBG, FOEWS, TFBG-SPR, and distributed optical fiber sensors based on Rayleigh scattering offer a vast range of possibilities for external and embedded applications in energy storage devices including lithium-ion batteries, sodium-ion batteries, silicon-carbon-based batteries, lithium-sulfur batteries, fuel cells, etc. They play an important role in monitoring battery stress-strain, temperature, electrolyte environment refractive index, material concentration, etc., detecting abnormal expansion, unusually high temperatures, deterioration of the electrolyte environment, lithium deposition, and alterations in the integrity of battery particles, which are crucial for improving the performance of energy storage devices and optimizing safety.

In addition to the several types of optical fiber sensors mentioned above, the application of new advanced optical fiber sensors, including dual-core (or multicore) fibers and photonic crystal fibers, has begun to receive attention. They can operate normally in extremely harsh environments and have resolution and accuracy beyond common sensors.

The several major types of optical fibers mentioned earlier can cause sensor failure due to rapid material degradation when exposed to high temperatures. When batteries or energy storage devices face thermal runaway, the instantaneous high temperature of hundreds of degrees Celsius will directly damage the foundation of the sensor, leading to sensor failure. Gold plated multicore microstructure optical fibers with special processing techniques can withstand extreme high temperatures up to 900 °C. This is of great significance for recording the accident process and conducting post-disaster accident reviews, which helps fundamentally improve the safety of energy storage devices. Studies have shown that mechanical parameters have a higher safety warning margin compared to electrical and thermal parameters. For batteries or energy storage devices, accurate monitoring of mechanical behavior can help excavate the safety status of batteries in the very early stages. Dual core optical fiber strain change sensors based on cross-sectional crosstalk changes have much better strain sensitivity than common FBG sensors [141, 142]; furthermore, when the multicore microstructure fiber Mach–Zehnder interferometer acts as a multichannel interferometer, research has shown that it has higher sensitivity while overcoming the cross-sensitivity problem between force and temperature.

**Fig. 11:**
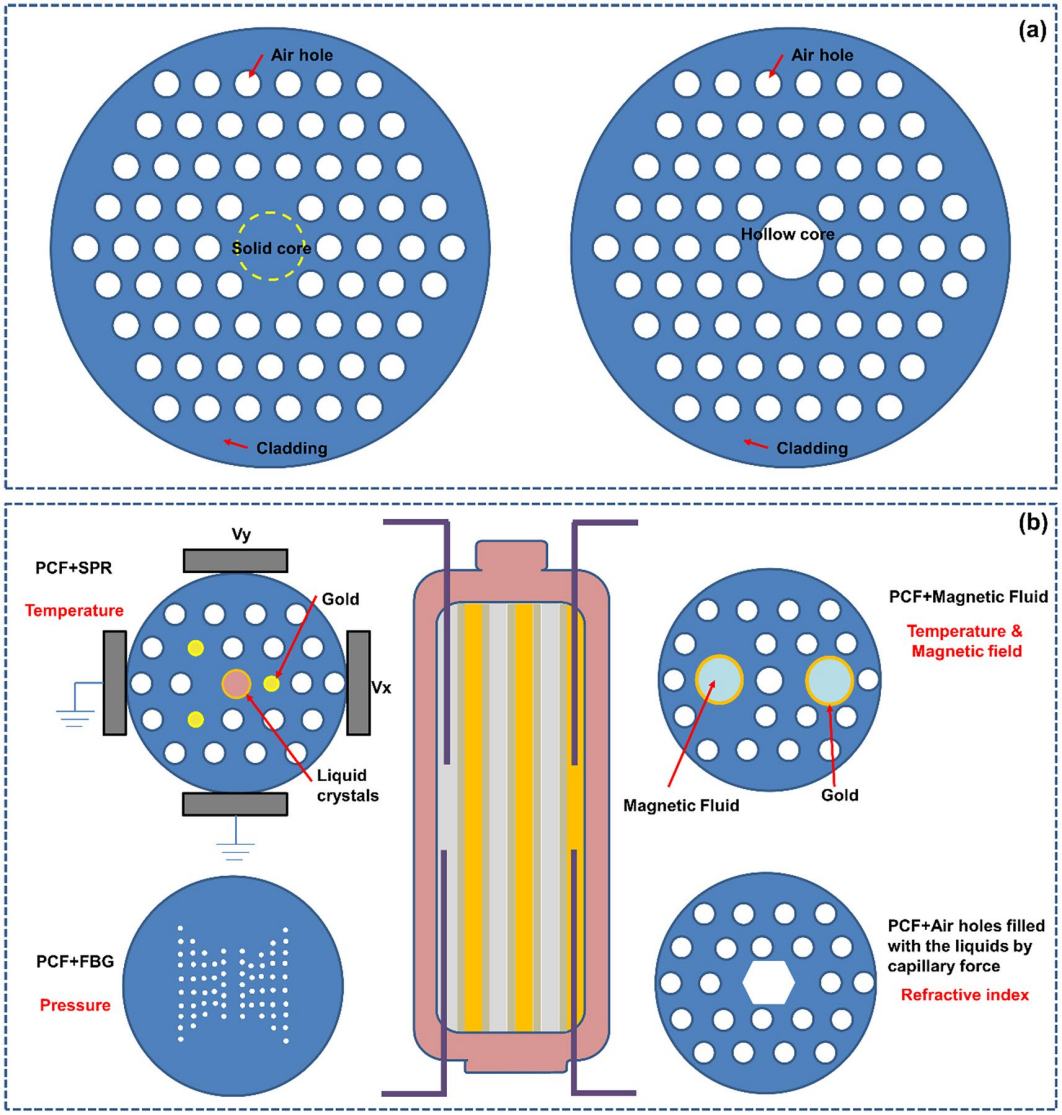
Future application prospects of advanced PCF optical fiber sensors. **a** PCF structure, left: solid core, right: hollow core. **b** The future application of different types of PCF in energy storage devices such as batteries will achieve comprehensive monitoring of the mechanical, electrical, thermal, and pressure of energy devices. Upper left: The combination of the PCF structure and the SPR principle, a temperature sensor is constructed using surface plasmon resonance liquid crystal to measure the core temperature of the device; Lower left: The combination of PCF and FBG, a high birefringence microstructure is fabricated using FBG to form a pressure sensor, enabling the measurement of the device internal core pressure; Upper right: The PCF hollow is filled with magnetic fluid to form a magnetic field-temperature composite sensor. By utilizing the sensitivity of magnetic fluid to magnetic field and temperature, the magnetic field and temperature are decoupled through structural design to achieve monitoring of the internal magnetic field and temperature of the energy device; Lower right: The combination of PCF and the measured liquid forms a refractive index sensor. The measured liquid is filled in the PCF hollow through capillary effect, and sensitive spectral changes are utilized to monitor the refractive index of the measured object

In our research, by carefully analyzing a vast array of literature and research studies, we have uncovered a remarkable optical fiber sensor—the photonic crystal fiber (PCF) optic physical sensor. This sensor can be seamlessly integrated with nearly all the advanced optical fiber sensors mentioned earlier, whether already implemented in batteries or potentially applicable, thereby expanding the range of sensing functions. It has a hollow or solid core, and the pores in the optical fiber are distributed around the core in different ways, and light is guided to propagate through the distribution of these pores (Fig. 11a). In addition, the propagation of light can be controlled by changing the distribution of holes and environmental changes [143]. PCF, with its unique advantages, is capable of sensing different physical parameters, such as temperature, pressure, strain, torsion, curvature or bending, electromagnetic field, and even refractive index. When used as a temperature sensor, it serves as a cutting-edge sensor, capable of exhibiting a sensitivity of 0.11 nm/°C in the temperature range of 100 to 700 °C [144], with extremely high measurement accuracy and high-temperature resistance performance; When used as a mechanical sensor, PCF can reach hundreds of pm per bar or tens of pm per με when combined with other types of spatial structured optical fiber sensor components. If applied to batteries or energy storage systems, it can easily monitor air pressure and material expansion; In addition, PCF has unique advantages over other optical fiber sensors in monitoring the torsion [145], bending, curvature [146] of the measured object; As mentioned earlier, PCF has hollow pores inside it, when the cladding air pores are filled with Fe_3_O_4_ nanofluid [147], its advantages as a highly sensitive magnetic field sensor become apparent. Its liquid concentration only needs 0.6 mg mL^−1^, which can achieve a magnetic field sensing accuracy of 242 pm (mT)^−1^. In the future, if it is applied to batteries or energy storage devices, we believe that there will be no escape for micro-internal short circuits in batteries; In terms of refractive index monitoring, unlike the local refractive index monitoring of TFBG, the internal pores of PCF can be filled with analytes. If applied to the electrolyte environment, it is also possible to achieve global electrolyte refractive index monitoring in the future, which has more practical value for monitoring the health and safety status of batteries.

PCF has a high degree of flexibility, which can be combined with many advanced fiber optic sensors or injected with specific components into pores to form colorful high-performance sensors, as shown in Fig. 11b and Table 4.

**Table 4:** Summary of multipurpose sensing for PCF and advanced optical fiber sensor

Structure	Parameter	Sensitivity	Refs.
PCF + SMF Tip interferometer by PCF spliced with SMF	Temperature	10 pm/°C	[148]
PCF + FP + SMF PCF-based Fabry–Perot interferometer, which includes an inline micro-cavity and is spliced with SMF	Temperature	12 pm/°C	[149]
PCF + SPR Plasma resonance-based gold nanowire liquid crystal PCF	Temperature	10 nm/°C	[150]
PCF + Isopropanol Isopropanol filled PCF long period grating	Temperature	1.356 nm/°C	[151]
PCF + long period gratings The combination of periodically tapered long period grating and PCF	Pressure/Strain	11.2 pm/bar/ -7.6 pm/με	[152, 153]
PCF + FBG High birefringence microstructure fiber based on Bragg grating	Pressure	33 pm/Mpa	[154]
PCF + Fe_3_O_4_ Fe_3_O_4_ nanofluid filling	Electromagnetic field	242 pm/mT	[147]
PCF + FP + CdFe_2_O_4_ CdFe_2_O_4_ as a magnetic fluid	Electromagnetic field	33 pm/Oe	[155]
PCF + SMF + Water-based ferromagnetic fluid Water-based ferromagnetic fluid EMG507	Electromagnetic field	16.04 pm/G	[156]
PCF + SPR Gold layer filled SPR	Electromagnetic field	−	[157]
Multi core flat optical fiber based on SPR	Refractive index	23,000 nm/RIU	[158]
PCF + Long Period Grating Surface long-period gratings incorporated D-shaped PCF	Refractive index	585.3 nm/RIU	[159]
PCF + filled analyte under test	Refractive index	2 × 10^−6^ RIU	[160]

At present, advanced optical fiber sensors, including PCF fiber, have certain applications in engineering, but there are few reports of their use in batteries and energy storage devices. Advanced fibers such as dual core fiber optic and PCF fiber optic have excellent performance, small size, and good system adaptability. We believe that they have broad application prospects in batteries and energy storage devices. In the future, with the development of technology, we believe that these advanced optical fiber sensors have the potential to achieve comprehensive sensing of mechanical, electrical, thermal, gas, and pressure in a single fiber.

### Optimization and Enhancement of Advanced Embedded Optical Fiber Sensors

With the great potential brought by reduction of the cost of energy storage batteries and traction batteries and the continuous improvement of the battery performance, the storage capacity of batteries will significantly increase. The quality, reliability, and lifespan of a battery are more crucial than ever, so an effective battery sensing system is crucial [50].

For the FBG sensor, which has a relatively high technical maturity and is widely used and studied in batteries, in the field of thermal sensing, the FBG sensor’s “reaction time” (signal rise time) is reduced by 28% compared with the general thermocouple sensor, and it has a higher resolution [7, 50, 161]. It has the characteristics of mechanical-thermal coupling measurement and has the function of single-optical-fiber multipoint measurement [50]. The key point is that it can stably work in a harsh battery environment without disturbing the battery performance [50]. These advantages endow the optical fiber sensor represented by an FBG with faster, more precise and deeper application value.

**Fig. 12:**
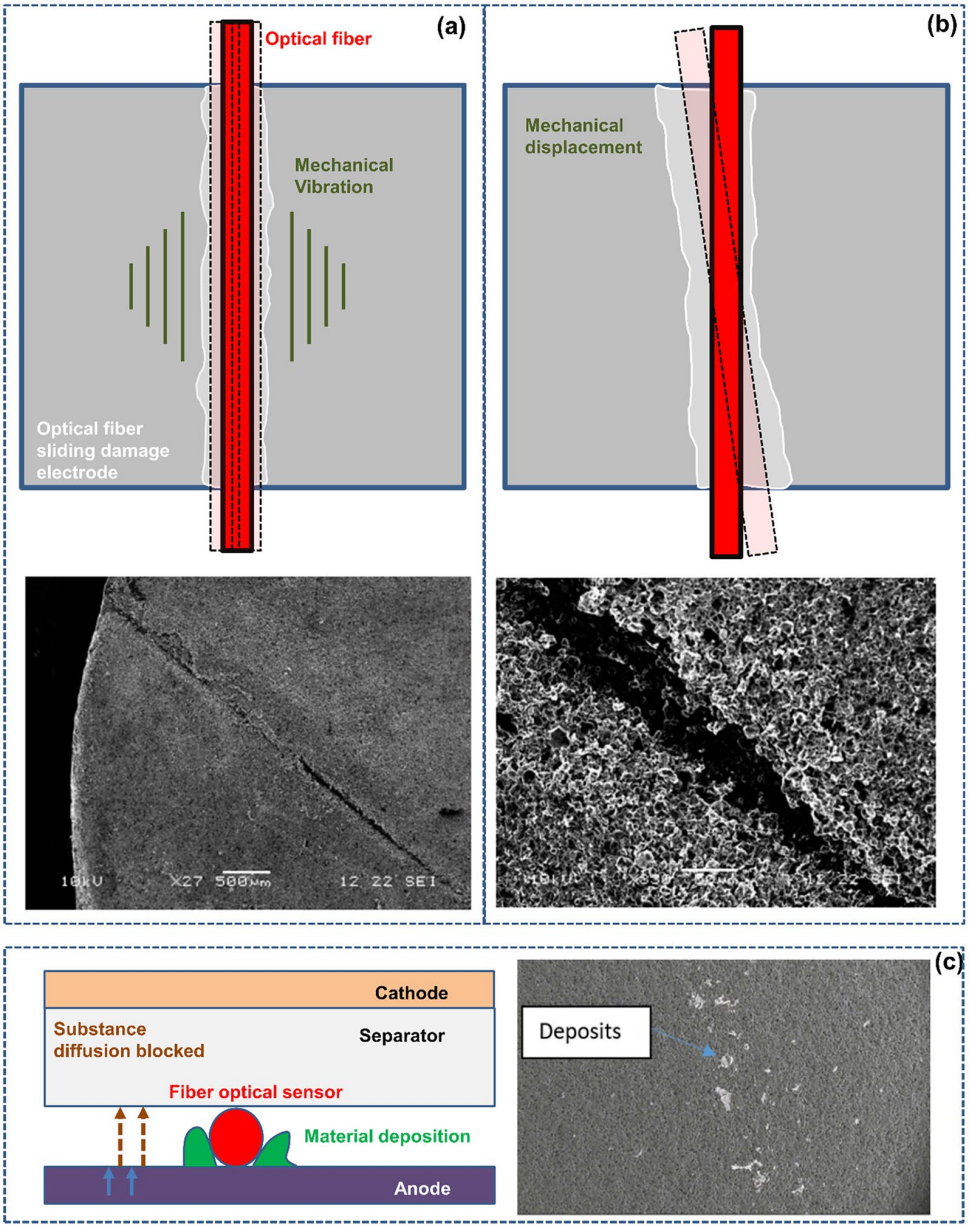
An electrode was damaged due to improper mechanical behavior of the optical fiber inside the battery. **a** The electrode surface is scratched due to mechanical vibration. Reproduced with permission from Ref. [139]. Copyright 2017 MDPI. **b** Material damage is caused by mechanical displacement. Reproduced with permission from Ref.[139]. Copyright 2017 MDPI. **c** The change in the electrode structure due to the optical fiber sensor leads to obstruction of material diffusion and material deposition. Reproduced with permission from Ref. [44]. Copyright 2022 MDPI

Despite all these advantages, the technological development experience indicates that the application of advanced optical fiber sensors in batteries may encounter challenges in the future. First, for lead-acid batteries [76, 162] with large shapes and sufficient internal space, the tiny size of the optical fiber sensor will not affect the battery performance. However, as shown in Fig. 12, the optical fiber sensor is still too harsh for thin electrodes in terms of technical details, which may affect the local integrity of active materials, resulting in sensing errors and even failure [139]. Second, improper fixation of the optical fiber will cause its unrestrained movement, when the harm is relatively small, the thin optical fiber is easily deflected due to the surface tension of the surrounding liquid materials, thus disturbing the sensor measurement [163], and in severe cases, optical fibers can scratch the active material, causing battery performance degradation or directly threatening battery safety. Third, the existence of an optical fiber in the electrode will damage the lithiation near the sensor, hinder diffusion of lithium ions, and create an effect similar to the diffusion blocking behavior caused by defects [44].

In addition to the above issues, there are still many obstacles to the embedded application of optical fibers in batteries. First, thin optical fibers are easy to be damaged or broken in the face of improper operation, which may cause signal loss, or even lead to internal short circuit contact and thermal runaway. Then, additional sealing measures are also an indispensable aspect of the application of optical fibers in batteries.

In the previous part, we summarized the technical challenges at the level of individual batteries in the embedded application of optical fibers in batteries, including issues such as the damage of foreign objects (fibers) to electrode materials, the impact of the deposition of substances around the fibers on monitoring accuracy, and the potential risk of the fragile fibers piercing the battery and causing thermal runaway. When we move up to the module level, new problems will arise. For example, although reflection-based optical fiber sensors can reduce the difficulty of sealing to some extent (the fiber only needs to be embedded without considering its emergence), at the module level, the advantages of distributed or quasi-distributed sensing are compromised when one single embedded fiber is used. Continuous interconnection of fibers with battery modules will pose significant challenges in the manufacturing process. Furthermore, in Part 2, we introduced several promising optical fiber sensors that can ensure the stable operation of fibers in batteries to some extent without excessively interfering with the normal operation of the battery. However, many studies have only verified for a few hundred hours or a few hundred cycles. Commercial 3C batteries, traction batteries, or energy storage batteries typically need to operate stably for several years or a decade. The batteries will undergo thousands of cycles. Therefore, there is currently no comprehensive research on whether optical fiber sensors can guarantee stable operation within batteries for such a long time. Hence, there is uncertainty about the long-term stability of optical fiber sensors over the years.

In that case, it is currently difficult to solve all technical problems by simply placing optical fibers inside the battery. Therefore, at this stage, applying a certain external structure to the optical fibers can optimize resolution and accuracy while providing a certain protective effect for the battery. Moreover, the external installation of optical fibers and the combination of traditional electrical and thermal sensors for batteries can be an optional alternative method that significantly improves existing monitoring technology. By utilizing the sensitivity of optical fibers to mechanical and thermal parameters, adding additional input parameters in SOC estimation can effectively improve the accuracy of SOC estimation; In terms of battery temperature monitoring, the use of optical fibers can enhance the coverage of temperature monitoring, allowing temperature monitoring to penetrate into each individual battery.

Therefore, to realize practical application of advanced optical fiber sensors in future batteries, several important issues need to be considered. Undoubtedly, in terms of basic theory, the primary problem is that the sensor must be able to adapt to the working environment and thoroughly characterize, optimize and calibrate the constitutive parameters such as the battery temperature, stress, strain and chemical composition (i.e., the selection of battery materials, additives and electrolytes). Then, in terms of engineering technology, to avoid damage to the sensor or electrode, the distribution and installation of the optical fiber in the electrode should be optimized to avoid affecting battery lifespan. At the same time, the optical fiber should be tightly sealed near the input and output terminals of the battery to prevent battery degradation due to exposure to water and oxygen. Additionally, in terms of production cost, simplifying and reducing the cost of optical equipment and components is the ultimate guarantee for the application of the technology [43, 164–166].

### Advanced Optical Fiber Sensors Drive the Development of Future Smart Batteries

Higher capacity, faster charging-discharging rate, and stronger safety are the “Olympic Motto” of batteries. A higher capacity brings a greater risk of thermal runaway of the battery. The existing BMS cannot carefully control each single battery. An aging battery will drag down the overall system performance, much like how the capacity of a bucket depends on its shortest board. A “fool-type” battery has no reaction ability in the face of extreme fast charging and other abuse working conditions. It can only passively accept the impact of a large current or external obstacles and is prone to accelerated battery aging, lithium plating or even dendrite formation due to an abnormal internal/external environment [167]. Therefore, various complex working conditions will lead to a sharp decline in the battery safety performance. The common standard BMS generally only has the ability to monitor the electrical-thermal parameters (current, voltage and temperature) of the battery, and this function is based on the module level. The BMS cannot provide information about the changes in the electrochemical state of the internal battery materials [167].

**Fig. 13:**
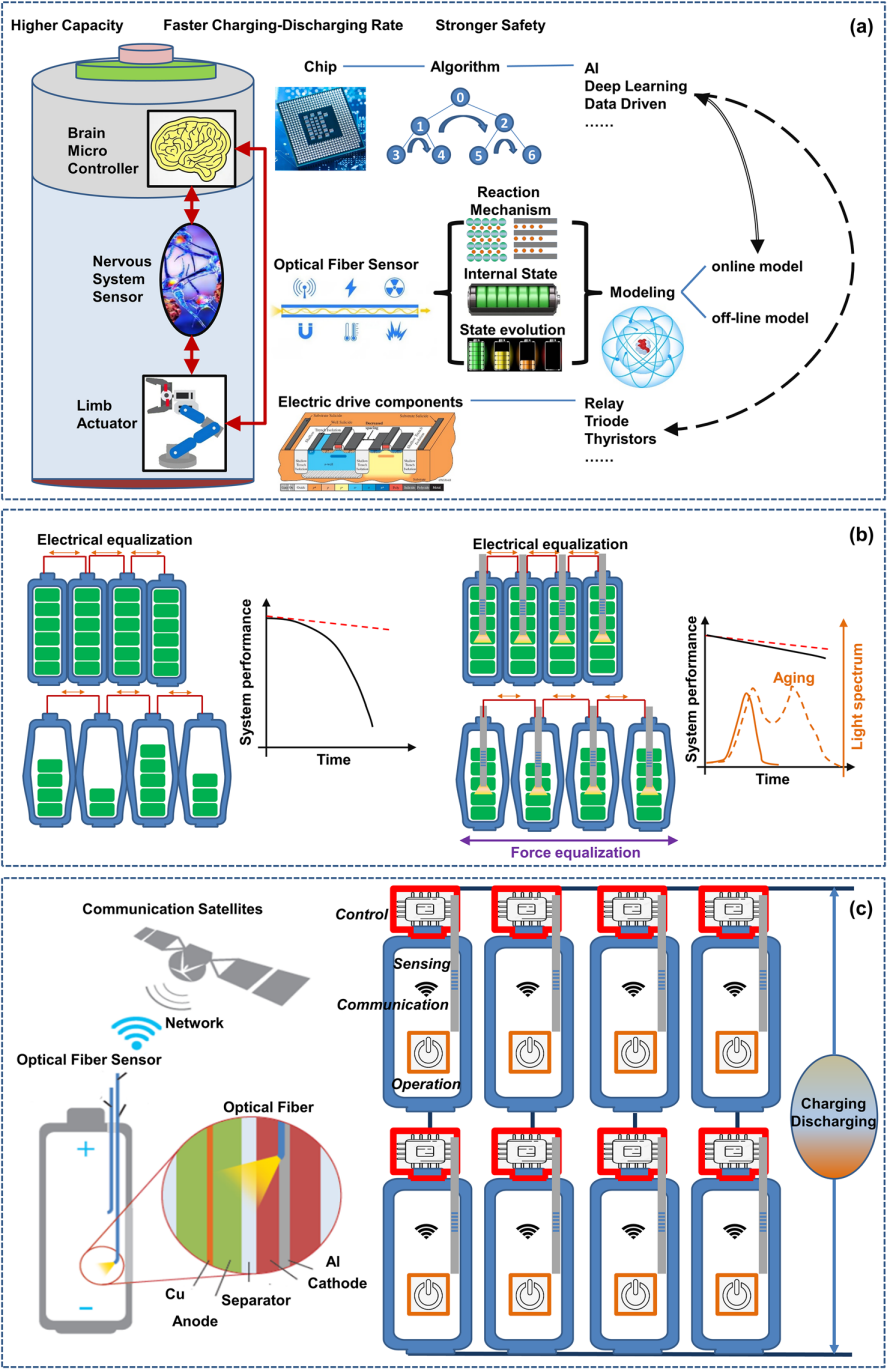
Advanced optical fiber sensors and microcontrollers in the future will help batteries be more powerful, safe, and intelligent. **a** The sensor and controller enable the battery to have self-sensing and self-control abilities. **b** In the future, it is possible to significantly promote system performance through embedded optical fiber sensor-smart batteries by enhancing the mechanical consistency. **c** Advanced smart batteries in the future will promote the progress of battery systems toward refined management. Reproduced with permission from Ref. [3]. Copyright 2020, published by EU

An advanced multisource parameter sensor will endow the battery with a “neural system”, a high-performance embedded microcontroller will equip the battery with a “brain”, and a microcontrollable actuator equipped with “limbs” for the battery (as shown in Fig. 13a. In the future, with the maturity of embedded sensors, miniature and reliable sensors can obtain more information about the internal working status of the battery, such as reaction mechanisms, internal states, and the evolution of battery states. On the basis of a deeper understanding of the internal core state of the battery by embedded optical fiber sensors, advanced algorithms such as digital twin models will help predict the performance of the battery more accurately and determine the optimal parameter performance of the battery in advance, thereby helping to capture the electrochemical performance of the battery and optimize lithium-ion batteries [168]. The development of sensors and familiarity with the internal mechanisms of batteries can encourage researchers to establish more refined and accurate models, both online and offline, which has the effect of “feeding back” the progress of battery technology. With the progress of intelligent algorithms, deep learning, data driven and even artificial neural network technology, the battery will have a pioneering “thinking ability” [169]. With the support of sensors and microcontrollers, both internal and external actuators of the battery can comprehensively control the operating status of the battery, including intelligent adaptation, self-balancing, and self-disconnection, to maintain system performance at the global optimum. Further technological progress will inevitably enable the battery to have a self-repairing function [170]. Then, a battery with perception, thinking, control and even self-healing abilities will overturn our cognition of the traditional battery [171–178].

As an intermediate development process, advanced optical fiber sensors have unique effects in enhancing individual consistency at the system level at this stage. Current researches show that the preload applied to the battery will have an impact on the electrical and thermal performance of the battery. In some battery systems, in order to maximize the energy density of the entire system, the stacking between batteries becomes increasingly tight, and the preload that the battery bears increase. As the battery continues to serve, the expansion of the battery will be inevitable, which will lead to a situation where middle batteries and the batteries on both sides bear significantly inconsistent external forces. As the cycles number increases, the electrical, thermal, and mechanical properties of the battery will simultaneously deteriorate the consistency between individuals. The application of advanced optical fiber sensors will open up the feasibility of monitoring the mechanical consistency state, and we believe that it is highly possible to achieve relevant monitoring effects through peak splitting of spectral signals. When mechanical consistency monitoring becomes possible, it is possible for the battery system to simultaneously adjust the consistency between individual through electric energy splitting and preload adjustment, enhancing the effectiveness of the battery system, as shown in Fig. 13b.

At present, there are still problems with optical fiber sensing, such as low fracture strength, poor toughness, and difficulties in decoupling cross-sensitive parameters. Therefore, the combination of optical fiber sensors and traditional sensors will be an effective transition method, which can simplify the technical difficulty and reduce costs while solving the problem of sensing and monitoring. The combination of optical fiber sensors with thermocouples or thin film strain gauges will effectively solve the problem of temperature-stress cross-sensitivity. The combination of optical fiber sensors with Hall electromagnetic field sensors will help to locate and warn micro-internal short circuit batteries in advance. Such combination will assist in the continuous development and optimization of optical fiber sensors. In addition to quantitative monitoring sensing at the data level, there is also the possibility of complementary advantages between general monitoring sensing technology and optical fiber sensing technology. The combination of thermal imaging technology and optical fiber sensing technology helps to accurately locate faulty batteries from the surface dimension to the point dimension [114]. The audio frequency sensing function of optical fiber sensors can accurately locate the battery rupture audio recorded by the microphone and timely locate the source of the fault [116]. The organic combination of advanced optical fiber technology and traditional sensing technology will give the two a new look, promoting the rapid development of battery safety perception and smart battery technology.

With the development of future technology, the use of advanced embedded optical fiber sensors combined with a low-cost and high-performance single chip controller will greatly reduce the computing power requirements of the central controller and even eliminate the need for the central controller to achieve robust and reliable distributed sensing control [7]. A further development is that each individual smart battery equipped with the advanced embedded optical fiber sensor, the intelligent algorithms and the actuator is equivalent to a minimum battery system. With the help of various embedded sensors, advanced algorithms, and advanced actuators, it can sense the working status of itself and all other batteries, thereby dynamically adjusting its own working status. The deterioration of one or more batteries will no longer be at the cost of sacrificing the performance of all batteries, and this greatly reduces the requirement for consistency. Currently, research on smart batteries is still in its infancy. The EU [3] and the Ministry of Industry and Information Technology of China [179] have proposed roadmaps for the development of smart batteries in the past two years. Advanced smart batteries will play a significant role in battery self-management and adaptation as shown in Fig. 13c. Before the revolutionary breakthrough in battery safety and performance, the development of smart batteries will be a strategic “detour”, but this does not mean that smart batteries are a “transitional technology” on the road to the essential improvement of the battery performance. The development of smart battery technology will enable the battery to return to the original “know itself” concept.

At present, the research on smart batteries is largely fragmented. Researchers conduct studies independently in areas such as embedded sensors, embedded microcontrollers, state estimation algorithms, artificial neural network algorithms, advanced repair materials and other fields. There is no systematic overall team conducting comprehensive research on smart batteries, which would involve the cross-cooperation of engineering, computer, materials and other disciplines. This puts strict requirements on the research team. The development of smart battery technology will not be smooth. There are many problems to be solved. The smart battery technology route will show an upward spiral trend until all technical and scientific problems are solved.

## Conclusion

The internal working state and working mechanism of energy storage components such as batteries are still a “black box” for researchers, and correctly opening the “black box” plays a crucial role in improving the performance of the energy storage system. Advanced optical fiber sensors play an important role in the future development of batteries and other energy storage devices. Because of their small size and excellent chemical stability, they can maintain long-term stability in a narrow and harsh battery environment. Optical fiber sensors can serve as a valuable tool for the “physical examination” of batteries. Using different principles, including the mechanical-thermal expansion effect, evanescent wave effect, surface plasmon wave effect or Rayleigh scattering effect, advanced optical fiber sensors can carry out all-round and cross-scale monitoring of batteries from external to internal, from the macroscale to microscale, and from the electrode interface to the electrode interior even chemical elements. Surprisingly, such sensors can enable the battery to have a pioneering “nerve”. Using the relevant mechanism, an optical fiber sensor can enable a battery to perceive stably and reliably the phase change process, parasitic reaction characteristic process, electrolyte internal environment degradation, safety performance evolution and global force-thermal characteristics. The battery “evolution” has created a “nerve”, establishing a robust foundation for the future development of high intelligence of batteries. Advanced optical fiber sensors can be used not only in batteries but also in other energy storage systems, such as sodium-ion batteries, lithium-air batteries, supercapacitors, fuel cells and other new chemical energy sources.

Advanced optical fiber sensors have a “milestone” significance on the road to promoting battery intelligence. Embedded sensors will give batteries a groundbreaking ability to sense their internal state. It is a consensus in the academic community that the internal battery information can reflect the actual safety status earlier and more clearly. In addition, advanced optical fiber sensors have the ability to identify battery electrical, thermal, elemental redox, electrolyte quality evolution, interface state evolution, and local electrical characteristics, which will greatly enrich the current simple electrical-thermal sensing signals. Due to the availability of more, more accurate, and deeper information, we may gain a deeper understanding of the reaction mechanism in lithium-ion batteries and other energy storage systems. With the support of the reaction mechanism, battery models will become more precise, and algorithms based on pinpoint models will become more mature. Therefore, battery performance and safety evolution will be perceived more accurately and at an early stage, we will have the ability to fully understand batteries.

Optical fiber sensors have the advantages of small size, fast response, high accuracy, high sensitivity, simple principle, multiple measurable signals, mature technology, and low cost in use. However, if embedded in batteries, their fragile fibers with radial dimensions slightly larger than the electrode thickness, fiber barriers to material transmission, sealing issues, and fiber stability issues in electrolyte environments become challenges in applying optical fiber sensors in batteries. In addition, the changes in production methods caused by the embedding of optical fibers into batteries will lead to an increase in costs, which are all issues that need to be addressed. We believe that strengthening the toughness and strength of optical fibers during manufacturing can fundamentally solve the problem of fiber optic fragility; For the problem of its large radial size, taking FBG as an example, the diameter of the fiber core is generally on the order of ten micrometers, and the thickness of the cladding and coating layer mainly affects the diameter of the fiber. In the future, it is entirely possible to find the optimal solution in terms of performance and radial size in technology; Engineering problems such as the sealing and the stability in electrolytes can fully meet both functionality and stability without excessively affecting production status through reasonable sealing methods and anti-corrosion coatings; Among the numerous problems, the possibility of optical fiber hindering material transmission is the most difficult theoretical and technical challenge that may exist. This requires joint research from the mechanism level to the material level and in the last the technical level, to minimize the obstruction of embedded components to battery material transmission and prevent sensors from becoming “harmful foreign objects”.

As mentioned earlier, the application of advanced optical fiber sensors in batteries will be a “double-edged sword”, which will bring about performance improvement but may threaten the battery safety. Such sensors can greatly improve the battery performance and will provide a “dimensionality reduction approach” for the current weak BMS. However, improper transmission and embedding of the sensor may easily cause lithium plating or even dendrite formation in the battery, which will threaten the battery safety due to thermal runaway. The development of advanced embedded optical fiber sensors and even smart batteries will certainly go through a “spiral” process, in which there will be “labor pains” and cost increases due to the instability of early products. However, with the continuous development of technology, we believe that advanced optical fiber sensors and smart batteries will certainly attract great attention in the future.
